# CHH hypermethylation contributes to the early ripening of grapes revealed by DNA methylome landscape of ‘Kyoho’ and its bud mutant

**DOI:** 10.1093/hr/uhae285

**Published:** 2024-10-14

**Authors:** Tong-Lu Wei, Yu-Tong Wan, Hai-Nan Liu, Mao-Song Pei, Guang-Qi He, Da-Long Guo

**Affiliations:** College of Horticulture and Plant Protection, Henan University of Science and Technology, Luoyang 471023, China; Henan Engineering Technology Research Center of Quality Regulation of Horticultural Plants, Luoyang 471023, China; College of Horticulture and Plant Protection, Henan University of Science and Technology, Luoyang 471023, China; Henan Engineering Technology Research Center of Quality Regulation of Horticultural Plants, Luoyang 471023, China; College of Horticulture and Plant Protection, Henan University of Science and Technology, Luoyang 471023, China; Henan Engineering Technology Research Center of Quality Regulation of Horticultural Plants, Luoyang 471023, China; College of Horticulture and Plant Protection, Henan University of Science and Technology, Luoyang 471023, China; Henan Engineering Technology Research Center of Quality Regulation of Horticultural Plants, Luoyang 471023, China; College of Horticulture and Plant Protection, Henan University of Science and Technology, Luoyang 471023, China; Henan Engineering Technology Research Center of Quality Regulation of Horticultural Plants, Luoyang 471023, China; College of Horticulture and Plant Protection, Henan University of Science and Technology, Luoyang 471023, China; Henan Engineering Technology Research Center of Quality Regulation of Horticultural Plants, Luoyang 471023, China

## Abstract

DNA methylation is a stable epigenetic mark that plays a crucial role in plant life processes. However, the specific functions of DNA methylation in grape berry development remain largely unknown. In this study, we performed whole-genome bisulfite sequencing on ‘Kyoho’ grape and its early-ripening bud mutant ‘Fengzao’ at different developmental stages. Our results revealed that transposons (TEs) and gene flanking regions exhibited high levels of methylation, particularly in ‘Fengzao’, attributed to CHH site methylation. Interestingly, the methylation patterns in these two cultivars showed distinct dynamics during berry development. While methylation levels of genes and TEs increased gradually in ‘Kyoho’ throughout berry development, ‘Fengzao’ did not display consistent changes. Notably, ‘Fengzao’ exhibited higher methylation levels in promoters compared to ‘Kyoho’, suggesting that hypermethylation of promoters may contribute to its early ripening phenotype. Integration of methylome and transcriptome data highlighted differentially methylated genes (DMGs) and expressed genes (DEGs) associated with secondary metabolite biosynthesis, with 38 genes identified as potential candidates involved in grape berry development. Furthermore, the study identified a jasmonate-induced oxygenase gene (*JOX1*) as a negative regulator of ripening in *Arabidopsis* and grapes, indicating that hypermethylation of *JOX1* may play a role in the early ripening of ‘Fengzao’. Overall, our findings provide insights into the distinct DNA methylation patterns during grape berry development, shedding light on the epigenetic regulatory mechanisms underlying the early-ripening bud mutant.

## Introduction

Cytosine DNA methylation is a conserved epigenetic mark significantly associated with chromatin functions in plants and mammals. DNA methylation plays important roles in DNA recombination, gene imprinting, and genome stability, affecting various biological processes, such as plant growth, fruit development, flower differentiation, stress responses [1–8], human aging [9], and cancer development [10, 11]. DNA methylation in plants occurs in the symmetric CG and CHG sequence contexts, as well as the asymmetrical CHH sequence context (with H representing A, C, or T) [3, 4]. In *Arabidopsis*, the methylation of CG and CHG (mCG and mCHG) are maintained during the DNA replication process, which requires DNA methyltransferases, while mCHH is maintained by chromomethylase and domain rearranged methyltransferases through the RNA-directed DNA methylation (RdDM) pathway [12, 13].

Fruit development is a complex biological process affected by multiple internal factors, including the accumulation of bioactive compounds, modified composition and texture, susceptibility to pathogens, and joint participation of phytohormones [14–17]. Recent studies demonstrated that fruit development is also affected by epigenetic modification, during which the dynamic changes of DNA methylation are crucial for the ripening of both climacteric and non-climacteric fruits [18, 19]. The hypermethylation of the SBP-box transcription factor inhibited fruit ripening in tomato [20]. Further research showed a lower level of genome-wide DNA methylation in tomato during fruit development and that treatment with a methyltransferase inhibitor (5-azacytidine) led to an early-ripening phenotype [21]. The expression levels of methyltransferase genes *SlMET*, *SlDRM1L1*, *SlDRM5*, and *SlMET3L* and demethylase gene *SlDML* were significantly altered during tomato fruit development [22]. Knockout of the DNA demethylase gene *SlDML2* resulted in the hypermethylation of tomato, which led to the decreased expression of ripening-related genes and delayed fruit ripening [19, 23]. DNA methylation in apple plays a vital role in the formation of bud mutants and anthocyanin accumulation [24, 25]. Moreover, in non-climacteric fruits, the RdDM pathway was down-regulated during strawberry development, and DNA methylation inhibitors promoted strawberry ripening [26]. By contrast, during the development of sweet oranges, DNA demethylase genes were down-regulated, leading to increased DNA methylation levels, and treatment with DNA methylation inhibitor delayed fruit ripening [18]. Therefore, DNA methylation patterns vary between climacteric and non-climacteric fruits and among different fruit species of non-climacteric fruits (e.g., strawberry and sweet orange). However, the epigenetic regulation of DNA methylation on fruit development and ripening remains elusive.

Grape (*Vitis vinifera* L.) has a long history of cultivation and is one of the most important cultivated fruit crops worldwide [27]. Previous researches have demonstrated that hormones and reactive oxygen species (ROS) play a role in promoting grape ripening [28–30], and studies have reported that abscisic acid (ABA) could induce DNA methylation alteration in ripening-related genes in grapes [31]. Recent studies have unveiled the importance of methylation in grape berry development and ripening. DNA methylation served to suppress the expressions of genes linked to berry development, and 7 methyltransferase genes and 3 demethylation genes were differentially expressed during berry development [32]. Treatment with 5-azacytidine (a methylation inhibitor) on ‘Kyoho’ berries resulted in changes in methylation levels of genes, impacting precursor mRNA alternative splicing and gene expressions [33]. Whole-genome analysis of grapes has revealed that epigenetic modifications in genes and their flanking regions significantly influence gene expression, with a noted negative correlation between gene body methylation levels and average expression levels [34]. These findings suggest that methylation plays a crucial role in the processes of grape berry development and ripening.

‘Kyoho’ is a famous grape cultivar—from the hybridization of *V. vinifera* and *Vitis labrusca*—that ripens in early or middle July in China [27]. It is mainly consumed as table grapes in many Asian countries due to its excellent quality. ‘Fengzao’ is the early-ripening bud mutant of ‘Kyoho’, discovered previously in our laboratory, that ripens approximately 30 days earlier than ‘Kyoho’ [35]. The genetic backgrounds of ‘Fengzao’ and ‘Kyoho’ were highly similar based on the histological investigation [36], molecular marker analysis [36], and transcriptome analysis [37], suggesting the potential functions of epigenetic modifications on the early ripening of ‘Fengzao’, further confirming the involvement of DNA methylation in grape berry development. In this study, we conducted whole-genome bisulfite sequencing (WGBS) on ‘Kyoho’ and ‘Fengzao’ grape berries at various developmental stages to investigate the relationship between DNA methylation and berry development or ripening. Our findings provided a detailed understanding of the fluctuating patterns of DNA methylation throughout grape berry development, shed light on the epigenetic regulation mechanisms influencing the early-ripening process of ‘Fengzao’, and pinpointed potential genes involved in fruit development and ripening.

## Results

### Global view of the DNA methylomes in ‘Kyoho’ and ‘Fengzao’

**Figure 1:**
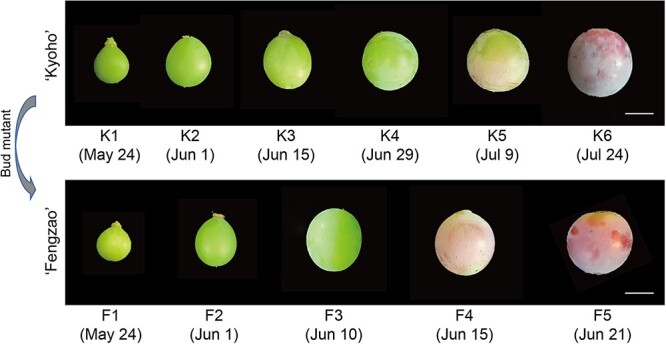
Berry images of ‘Kyoho’ and ‘Fengzao’ at different developmental stages. The sample name and the sampling date are shown below. Bar = 1 cm.

Whole-genome bisulfite sequencing (WGBS) was conducted on the berries of ‘Kyoho’ cultivar and its bud mutant ‘Fengzao’ at different developmental stages [37]. ‘Fengzao’ berries, ripening 33 days earlier than ‘Kyoho’ (Fig. 1), were sampled at five developmental stages (F1-F5), while ‘Kyoho’ berries were sampled at six stages (K1-K6) according to the E-L systems (Table 1) [38]. Each sample had two biological replicates and was sequenced with an average coverage of 10×, resulting in over 110 million clean reads, with over 60% mapped to the reference genome and over 98% cytosine (C) conversion rate (Table S1, Fig. S1). Methylation levels were calculated based on C base reads coverage, with analysis of C number and C coverage percentage at different sequencing depths (Table S2). The C coverage percentage for each sample at a 5× sequencing depth ranged from 21.99% to 68.14% (Table S2).

**Table 1:** Sampling information of naturally developing grape berries of ‘Kyoho’ and ‘Fengzao’

No.	Developmental stage	‘Fengzao’		‘Kyoho’		Berry trait
		Date	Sample name	Date	Sample name	
1	E-L31	5.24	F1	5.24	K1	pea-size berries
2				6.1	K2	
3	E-L32	6.1	F2	6.15	K3	berry touching
4	E-L33	6.10	F3	6.29	K4	hard green berries
5	E-L34	6.15	F4	7.9	K5	starting to soften
6	E-L35	6.21	F5	7.24	K6	véraison

**Figure 2:**
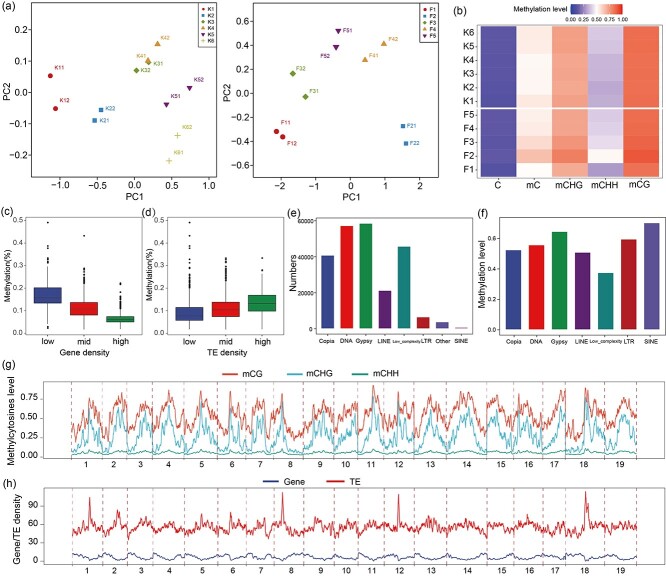
DNA methylation for each sample of ‘Kyoho’ and ‘Fengzao’. (a) PCA (Principal component analysis) results based on whole-genome bisulfite sequencing in ‘Kyoho’ (left) and ‘Fengzao’ (right). F: “Fengzao,” K: ‘Kyoho’. F1–F5 and K1–K6 indicate samples at different developmental stages in ‘Fengzao’ and ‘Kyoho’, respectively. Different samples are represented by different symbols, with each set of two biological replicates represented by the same symbol. (b) Heatmap showing the methylation levels of different types (C, mC, mCHG, mCHH, and mCpG) in each sample of ‘Kyoho’ and “Fengzao.” “C” and “mC” are the average methylation levels of unmethylated cytosine and methylated cytosine, respectively. The heatmap shows detailed data on the average methylation levels in different contexts. (c,d) Average DNA methylation levels of genes (a) and TEs (b) with low, middle, and high density. (e) Number of each of the seven representative types of TEs in the grape genome. (f) The average DNA methylation levels of the seven TE types. (g) The distribution of DNA methylation levels across the three sequence types (mCG, mCHG, and mCHH) across the chromosomes in ‘Kyoho’. (h) The distribution of gene and TE densities across the chromosomes in ‘Kyoho’.

Principal component analysis (PCA) showed that the two replications for all samples were clustered together and that samples from different developmental stages were clustered into different groups, indicating consistency across the two biological replications (Fig. 2a). The average DNA methylation levels for each sample were calculated (Fig. 2b, Table S3). There were obvious differences in mC in different samples, especially in “Fengzao,” with F1 and F2 samples showing the lowest and highest mC, respectively. When comparing methylation levels between ‘Kyoho’ and ‘Fengzao’ at the same developmental stages, the mC at F1 in ‘Fengzao’ was lower than the mC at K1 in ‘Kyoho’. The mC was higher in ‘Fengzao’ than in ‘Kyoho’ during development (F2, F3, K2, K3, K4). There were no obvious differences between the two cultivars during the late developmental stages (F4, F5, K5, K6) (Fig. 2b). A comparison of methylation levels under the sequence contexts of CG, CHG, and CHH (mCG, mCHG and mCHH) showed that, in all samples, mCG was relatively high, whereas mCHH was low (Fig. 2b).

Cytosine methylation levels vary across different sequence sub-contexts, including CG, CHG, and CHH. In this study, we examined the densities and methylation levels of each sub-context (CG, CHG, and CHH) along the chromosomes of ‘Fengzao’ to explore the impact of sequence variations on methylation levels. Our analysis revealed no significant differences in methylation levels among different CG sequences (CGA, CGC, CGG, and CGT). However, we observed that CCG motifs exhibited lower methylation levels compared to CAG and CTG within the CHG sub-context. Additionally, within the CHH sub-context, CAA and CTA sequences displayed higher methylation levels than other sequence sub-contexts (Fig. S2, S3).

### Transposons (TEs) are highly methylated compared to genes

In plants, transposons (TEs) are highly methylated regions that play a vital role in the regulation of gene expression [39]. In this study, the DNA methylation levels of TEs were specifically evaluated to further explore DNA methylation in grapes. Analysis of the statistics of gene or TE density and methylation levels showed that methylation levels were negatively correlated with the gene density because chromosome regions with higher gene density had relatively lower methylation levels (Fig. 2c). By contrast, TE density and methylation levels were positively correlated, as chromosome regions with higher TE density had relatively higher methylation levels (Fig. 2d), indicating that TEs were highly methylated compared to genes. We investigated different types of TEs in the grape genome to determine the relationship between TEs and DNA methylation. All TEs were divided into eight sub-groups, with relatively larger amounts for *Copia*, DNA, *Gypsy*, LINE, and low complexity (Fig. 2e). All sub-groups had relatively higher methylation levels except for the low complexity sub-group (Fig. 2f). Exploration of the gene/TE distribution and methylation levels across the chromosomes showed four obvious TE peaks in chromosomes 1, 8, 12, and 18, and the methylation levels at these TE peaks were correspondingly higher in both ‘Kyoho’ (Fig. 2g, h) and ‘Fengzao’ (Fig. S4), further confirming the hypermethylation of TEs in grapes.

### ‘Kyoho’ and ‘Fengzao’ show completely different DNA methylation patterns during berry development

**Figure 3:**
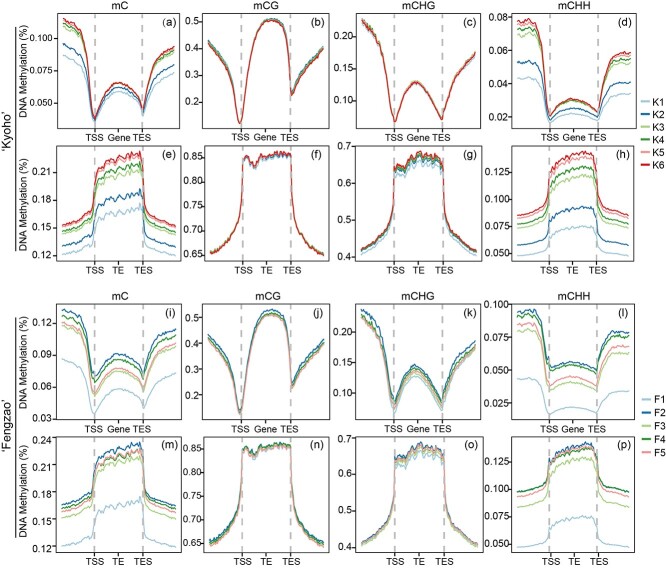
The DNA methylation levels across the adjacent regions of genes and TEs in ‘Kyoho’ (a–h) and ‘Fengzao’ (i–p) samples at different developmental stages. The x-axis shows regions of genes and TEs, including upstream regions of TSS (transcription start site), downstream regions of TES (transcription end site), and body regions (from TSS to TES). Samples at different developmental stages are represented by different colored lines.

The average methylation levels for genes and TEs were calculated separately to compare the DNA methylation changes during berry development between ‘Kyoho’ and ‘Fengzao’ (Fig. 3). Analysis of the methylation changes in genes and TEs showed that the transcription start site (TSS) and the transcription end site (TES) were significantly dividing points. Specifically, in genes, total cytosine methylation levels (mC) were lowest at the region near the TSS and TES, and the flanking regions (upstream TSS and downstream TES) showed relatively higher mC levels than the gene body regions (from TSS to TES) (Fig. 3a–d, i–l). However, in TEs, the mC of the body regions was significantly higher than that of the flanking regions (Fig. 3e–h, m–p), supporting the previous result that TEs were highly methylated. When comparing methylation levels among different samples of ‘Kyoho’ and ‘Fengzao’, we found that mC exhibited obvious changes in different samples (Fig. 3a, e, i, m), mainly due to mCHH, as more significant differences among different samples were observed for mCHH than mCG and mCHG (Fig. 3d, h, i, p). Moreover, the change patterns of DNA methylation were completely different between ‘Kyoho’ and ‘Fengzao’. DNA methylation levels of genes and TEs in ‘Kyoho’ gradually increased throughout the developmental stages (from K1 to K6), with the lowest and highest levels detected at K1 and K6, respectively (Fig. 3a–h). However, for ‘Fengzao’, changes in DNA methylation levels at different developmental stages followed no regular trend, with the lowest levels obtained at F1 and highest levels at F2 (Fig. 3i–p). Collectively, these results demonstrated that ‘Fengzao’ underwent different methylation change patterns (especially for mCHH) during berry development compared with ‘Kyoho’, which might contribute to the early ripening of ‘Fengzao’.

### ‘Fengzao’ has higher promoter methylation than ‘Kyoho’ during berry development

**Figure 4:**
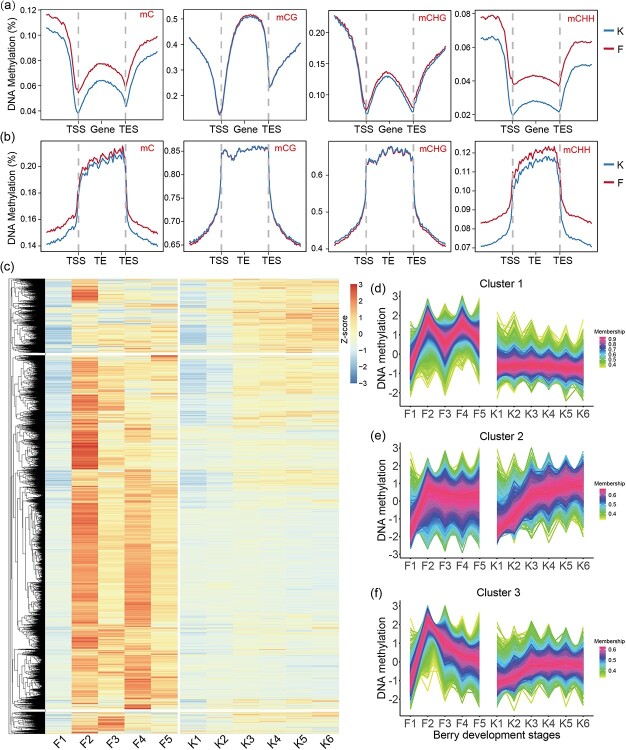
Analysis of promoter DNA methylation in ‘Kyoho’ and ‘Fengzao’. (a,b) DNA methylation levels across the regions of genes (a) and TEs (b) in ‘Kyoho’ (K) and ‘Fengzao’ (F). TSS: transcription start site, TES: transcription end site. (c) Heatmap showing the average methylation levels of promoter regions (2 kb upstream of TSS) in ‘Kyoho’ (K) and ‘Fengzao’ (F) samples at different developmental stages. DNA methylation values were normalized in row using the Z-score method. (d–f) Three clusters (Clusters 1–3) showing the methylation changes in different samples of ‘Kyoho’ (K) and ‘Fengzao’ (F), corresponding to the heatmap (c).

The average DNA methylation levels for genes and TEs were analyzed to compare the methylation differences between ‘Kyoho’ and ‘Fengzao’ (Fig. 4a, b). The mC of ‘Fengzao’ was higher than ‘Kyoho’ for both genes and TEs, especially for mCHH, and the methylation levels in promoter regions (upstream TSS) were relatively higher than in gene body regions (from TSS to TES) (Fig. 4a, b), suggesting that promoter regions might strongly affect gene expressions. Thus, we specifically analyzed the methylation levels of the 2-kb promoter regions upstream of the TSS of the genome-wide genes (Fig. 4c). As shown in the heatmap, except for F1, the promoter methylation levels of ‘Fengzao’ were higher than those of ‘Kyoho’ for most of the genes (Fig. 4c). All genes were divided into three clusters (1–3) based on the changes in promoter DNA methylation during the berry developmental periods (Fig. 4d–f). Analysis of the genes in cluster 1 showed sharp changes in the methylation levels of ‘Fengzao’ during developmental periods (F1–F5); however, ‘Kyoho’ exhibited steady methylation levels during all six developmental periods (K1–K6) (Fig. 4d). Analysis of cluster 2 genes showed that methylation levels of ‘Fengzao’ sharply increased from F1 to F2, and then remained relatively invariable, whereas those of ‘Kyoho’ showed an increasing trend from K1 to K6 (Fig. 4e). Analysis of cluster 3 genes showed that methylation levels of ‘Fengzao’ markedly increased from F1 to F2 before gradually decreasing from F2 to F5, whereas those of ‘Kyoho’ showed a gently increasing trend from K1 to K3, and then remained stable from K3–K6 (Fig. 4f). There were a total of 15 851 genes in cluster 3, which were mainly enriched in 19 GO terms including transcription regulatory region DNA binding, protein kinase activity, peroxidase activity and pectinesterase activity (Fig. S5). KEGG pathway enrichment analysis showed that a total of 5 pathways were enriched, including peroxidase, xyloglucosyl transferase, and SAUR family protein, etc. (Fig. S5). Overall, ‘Fengzao’ has higher methylation levels in promoter than ‘Kyoho’ during berry development.

### Statistics of differentially methylated cytosine sites (DMCs), regions (DMRs), and genes (DMGs)

The study further examined DNA methylation differences in various samples by analyzing differentially methylated cytosine sites (DMCs) in each comparison. Numerous hypermethylated (hyper) and hypomethylated (hypo) cytosine sites were identified in ‘Kyoho’ and ‘Fengzao’ cultivars. In ‘Fengzao’, more DMCs showed hypermethylation, particularly for CHH, in comparisons F2 vs. F1 and F4 vs. F3, while more DMCs exhibited hypomethylation in comparisons F3 vs. F2 and F5 vs. F4 (Fig. S6). Conversely, ‘Kyoho’ had more DMCs of hypermethylation compared to hypomethylation in all comparisons, especially for CHH (Fig. S7). Furthermore, the comparison between ‘Kyoho’ and ‘Fengzao’ at the same developmental stages revealed that the CHH type had more DMCs than the CG and CHG types. Specifically, comparisons K3 vs. F2, K5 vs. F4, and K6 vs. F5 displayed more DMCs of hypomethylation than hypermethylation (Fig. S8). These identified DMCs could serve as valuable markers for key genomic sites involved in grape berry development.

**Figure 5:**
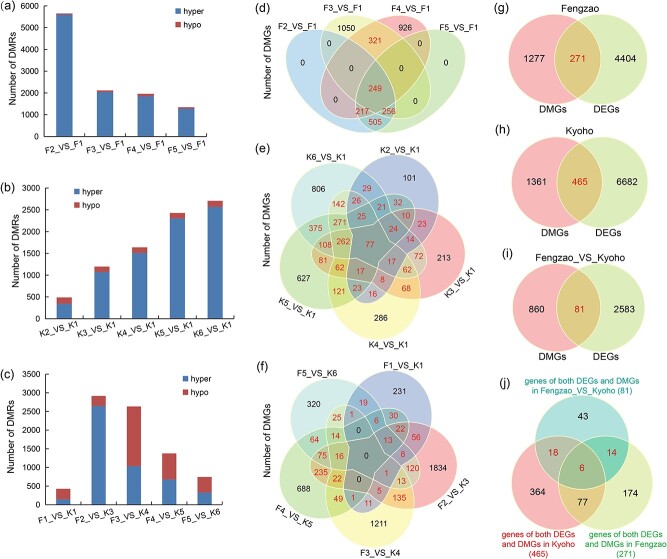
Statistics of differentially methylated regions (DMRs) and genes (DMGs). (a,b) Number of DMRs in ‘Fengzao’ (F) and ‘Kyoho’ (K) comparing with the first developmental stage. Hyper: hypermethylation; Hypo: hypomethylation. (c) Number of DMRs between ‘Fengzao’ and ‘Kyoho’ at the same developmental stage. (d–f) Venn diagrams showing numbers of DMGs between each comparison. Numbers in red font indicate key genes which were overlapped in two or more comparisons. (g–i) Venn diagrams showing conjoint analysis of DMGs and DEGs, based on the identified genes from Fig. d–f and Fig. S8. (j) Venn diagrams integrating the identified genes which are both DMGs and DEGs, based on the results from Fig. g–i.

Next, we analyzed the genomic distribution of differentially methylated regions (DMRs) in ‘Fengzao’ and ‘Kyoho’ during berry development. The number of DMRs in F2–F5 and K2–K6 was counted, respectively, in comparison with the first developmental stage (Fig. 5a, b). The DMRs gradually decreased from F2 to F5 in ‘Fengzao’ (Fig. 5a), while increased from K2 to K6 in ‘Kyoho’ (Fig. 5b). The majority of DMRs were hypermethylated (hyper) regions both in ‘Fengzao’ and ‘Kyoho’, suggesting that hypermethylation might occur during grape berry development. We also explored the DMRs between ‘Fengzao’ and ‘Kyoho’ at same developmental stages (F1 vs. K1, F2 vs. K3, F3 vs. K4, F4 vs. K5, and F5 vs. K6). F2 vs. K3 had much more hypermethylated DMRs than hypomethylated DMRs, and the DMRs of hypermethylation and hypomethylation in the other comparisons were almost same (Fig. 5c).

The genomic distribution of differentially methylated regions (DMRs) in ‘Fengzao’ and ‘Kyoho’ was analyzed during berry development. The number of DMRs in F2–F5 and K2–K6 was compared to the first developmental stage, with a gradual decrease in DMRs from F2 to F5 in ‘Fengzao’ (Fig. 5a) and an increase from K2 to K6 in ‘Kyoho’ (Fig. 5b). The majority of DMRs were found to be hypermethylated regions in both ‘Fengzao’ and ‘Kyoho’, indicating potential hypermethylation during grape berry development. Furthermore, DMRs between ‘Fengzao’ and ‘Kyoho’ at the same developmental stages were explored (F1 vs. K1, F2 vs. K3, F3 vs. K4, F4 vs. K5, and F5 vs. K6). F2 vs. K3 showed a higher number of hypermethylated DMRs compared to hypomethylated DMRs, while the comparisons in the other stages had similar numbers of hypermethylation and hypomethylation DMRs (Fig. 5c).

Differentially methylated genes (DMGs) were identified in ‘Kyoho’ and ‘Fengzao’, as well as between the two cultivars. The Venn diagrams illustrated the overlapping DMGs in multiple comparisons (Fig. 5d–f). A total of 1548 DMGs were found in ‘Fengzao’ (321 + 249 + 217 + 256 + 505) (Fig. 5d); 1826 DMGs in ‘Fengzao’ (Fig. 5e); and 941 DMGs in ‘Fengzao’ vs. ‘Kyoho’ (Fig. 5f). Additionally, we examined differentially expressed genes (DEGs) in ‘Fengzao’ and ‘Kyoho’ throughout berry development using previous transcriptome data [37] (Fig. S9) to determine if these DMGs were also DEGs. By conjoint analysis of DMGs and DEGs with Venn diagrams, we identified many candidate genes that were both DMGs and DEGs (Fig. 5g–i). Specifically, 271 and 465 genes were found to be differentially expressed and differentially methylated during berry development in ‘Fengzao’ and ‘Kyoho’, respectively (Fig. 5g, h), while 81 genes showed differential expression and methylation between ‘Fengzao’ and ‘Kyoho’ at the same developmental stage (Fig. 5i). A Venn diagram combining these sets of genes revealed that 38 (18 + 6 + 14) genes were both DEGs and DMGs during berry development and in the comparison between ‘Fengzao’ and ‘Kyoho’ (Fig. 5j). These 38 identified genes represent important candidate genes that may play a role in grape berry development and the early ripening of ‘Fengzao’.

### Promoters of genes on biosynthesis of secondary metabolites were hypermethylated during berry development

**Figure 6:**
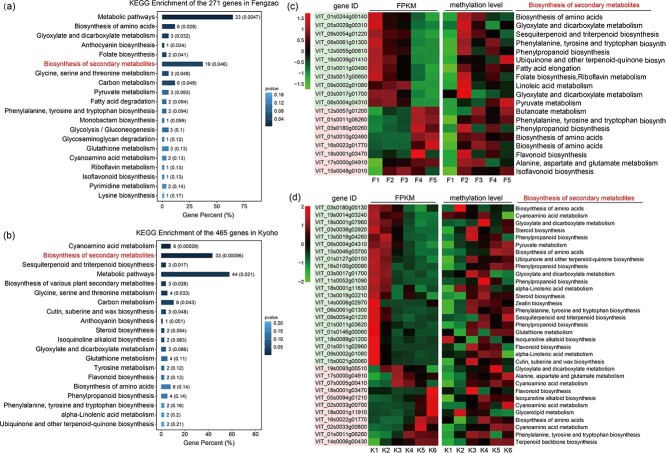
KEGG pathway enrichment analysis of the identified 271 and 465 genes in ‘Fengzao’ (F) and ‘Kyoho’ (K). (a,b) The results of KEGG pathway enrichment in ‘Fengzao’ (a) and ‘Kyoho’ (b). The bar graphs indicate gene percent (%), with the depth of the blue color showing p values. The numbers on the bars indicate the gene number, and the numbers in the parentheses indicate the p values. (c,d) The heatmaps showing the FPKM values and the promoter methylation levels of the 19 and 33 genes in the pathway of “Biosynthesis of secondary metabolites” in ‘Fengzao’ (c) and ‘Kyoho’ (d). The values are normalized in row using the Z-score method. The gene IDs in red and green background indicate up-regulated and down-regulated genes, respectively. The functions of these genes are exhibited on the right.

In the previous study, a total of 271 and 465 genes were identified as differentially methylated genes (DMGs) and differentially expressed genes (DEGs) during berry development in ‘Fengzao’ and ‘Kyoho’, respectively (Fig. 5g, h). To elucidate the functions of these genes, pathway enrichment analysis using the Kyoto Encyclopedia of Genes and Genomes (KEGG) was performed. The analysis revealed that the top enriched pathways were primarily associated with the biosynthesis of secondary metabolites. Specifically, pathways such as “Glyoxylate and dicarboxylate metabolism”, “Anthocyanin biosynthesis”, “Folate biosynthesis”, and “Pyruvate metabolism” were enriched in ‘Fengzao’ (Fig. 6a), while “Cyanoamino acid metabolism”, “Sesquiterpenoid and triterpenoid biosynthesis”, “Anthocyanin biosynthesis”, and “Steroid biosynthesis” were enriched in ‘Kyoho’ (Fig. 6b). Further analysis of the “Biosynthesis of secondary metabolites” pathway in ‘Fengzao’ and ‘Kyoho’ revealed 19 and 33 genes, respectively. Examination of FPKM values showed that during berry development, 11 genes were down-regulated and 8 genes were up-regulated in ‘Fengzao’ (Fig. 6c), and 22 genes were down-regulated and 11 genes were up-regulated in ‘Kyoho’ (Fig. 6d). Assessment of the promoter methylation levels of these genes indicated an overall increasing trend during berry development in both ‘Fengzao’ and ‘Kyoho’ (Fig. 6c, d). This suggests that the promoters of genes involved in the biosynthesis of secondary metabolites are hypermethylated during grape berry development, leading to differential gene expression that may impact the synthesis of secondary metabolites.

### The identified candidate genes hypermethylated in ‘Fengzao’ and involved in berry development

**Figure 7:**
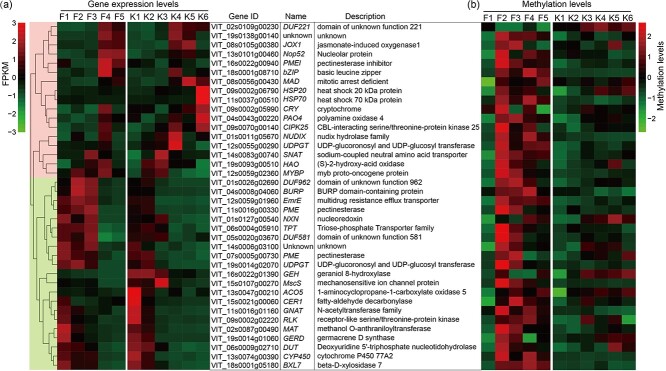
Gene expression levels (a) and promoter methylation levels (b) of the 38 candidate genes in ‘Fengzao’ (F) and ‘Kyoho’ (K) during berry development. The left heatmap (a) shows the FPKM values, and the right heatmap (b) shows the methylation levels. The values of FPKM and methylation levels are normalized within each row using the Z-score method. A total of 38 genes are clustered on the evolutionary tree based on gene expression levels, with a color scheme of red and green backgrounds indicating up-regulated and down-regulated genes during berry development. The gene ID, name, and description for each gene are presented in the center.

In the previous study, 38 candidate genes potentially involved in grape berry development and early ripening of ‘Fengzao’ were identified. Further analysis was conducted on the gene expression levels and promoter methylation levels of these genes (Fig. 7). The findings revealed that 17 genes exhibited an increasing trend in expression levels during berry development, such as *JOX1* (*jasmonate-induced oxygenase 1*), *PMEI* (*pectinesterase inhibitor*), *bZIP* (*basic leucine zipper*), *PAO4* (*polyamine oxidase 4*), *CIPK25* (*CBL-interacting serine/threonine-protein kinase 25*), and *HAO* (*S-2-hydroxy-acid oxidase*). On the other hand, 21 genes showed a decreasing trend during berry development, including *DUF962* (*domain of unknown function 962*), *BURP* (*BURP domain-containing protein*), *EmrE* (*multidrug resistance efflux transporter*), *PME* (*pectinesterase*), *NXN* (*nucleoredoxin*), *TPT* (*triose-phosphate transporter family*), *UDPGT* (*UDP-glucoronosyl and UDP-glucosyl transferase*), and *RLK* (*receptor-like serine/threonine-protein kinase*) (Fig. 7a). The comparison between ‘Fengzao’ and ‘Kyoho’ revealed similar trends in gene expression changes during berry development, but distinct expression levels were observed at the same developmental stage for the two cultivars (Fig. 7a). Notably, ‘Fengzao’ exhibited significantly higher promoter methylation levels compared to ‘Kyoho’ for most of these genes, particularly at stages F2, F3, and F4 (Fig. 7b and Fig. S10–S15). These results suggest that the identified candidate genes potentially play a role in grape berry development, and the differential promoter hypermethylation in ‘Fengzao’ may contribute to its unique gene expression patterns and early ripening compared to ‘Kyoho’.

### Hypermethylation of *JOX1* contributes to the early ripening of ‘Fengzao’

**Figure 8:**
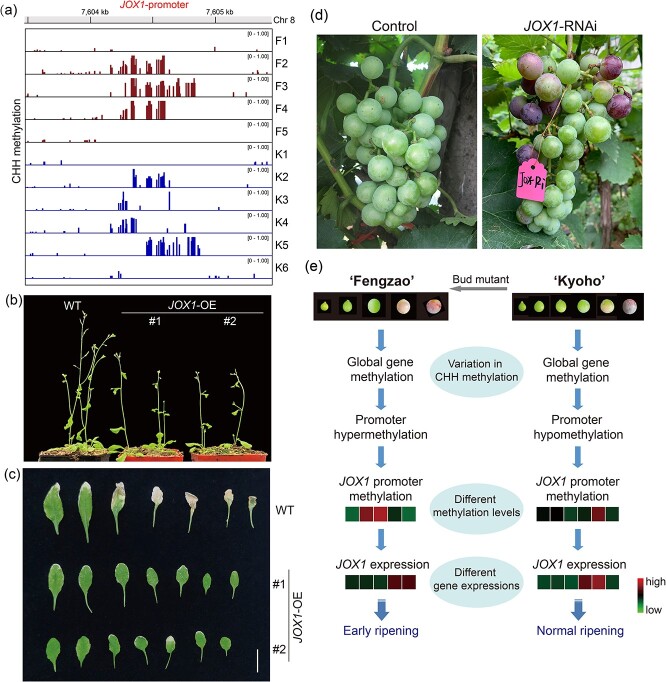
Hypermethylation of *JOX1* contributes to the early ripening of ‘Fengzao’. (a) Genome browser snapshot showing the CHH methylation of *JOX1* (VIT_08s0105g00380) promoter in ‘Fengzao’ and ‘Kyoho’ during berry development. (b) Plant phenotype of the 20-day-old *JOX1*-overexpression (*JOX1*-OE) and wild type (WT) *Arabidopsis* after same growth period under same conditions. (c) Leaf phenotype of the 35-day-old *JOX1*-OE and WT *Arabidopsis* plants. (d) Phenotype of grape clusters after infection with *JOX1*-RNAi (*JOX1*-pIR) and control (pIR) vectors at 45 days after full bloom (DAF). (e) A proposed model comparing the differences in mechanisms of berry development and ripening between ‘Fengzao’ and ‘Kyoho’. The heatmaps display FPKM and methylation levels, with red and green colors representing high and low values, respectively.

From the identified candidate genes, the gene encoding jasmonate-induced oxygenase, *JOX1*, was selected for investigating its role in early ripening. The promoter methylation levels of *JOX1* increased during berry development in both ‘Fengzao’ and ‘Kyoho’, peaking in the middle stages (F2, F3, F4, K2, K5) and decreasing in the early (F1 and K1) and late stages (F5 and K6) (Fig. 8a). This suggests that promoter CHH methylation plays a role in grape berry development. ‘Fengzao’ exhibited higher methylation levels compared to ‘Kyoho’, particularly at the F3 stage (Fig. 8a), indicating that hypermethylation may contribute to the early ripening of ‘Fengzao’. To further validate the role of *JOX1* in early ripening, the gene was overexpressed in *Arabidopsis*, resulting in delayed pod setting and leaf senescence compared to the wild type (Fig. 8b, c), suggesting that *JOX1* negatively regulates ripening. The results were further verified in ‘Kyoho’ grape berry by the pIR system [40], showing that knockdown of *JOX1* gene expression (*JOX1-*RNAi) promoting grape ripening in comparison with the empty vector control (Fig. 8d, Fig. S16). Based on these findings, we preliminarily summarized the mechanisms underlying the early ripening of ‘Fengzao’ (Fig. 8e). Throughout berry development, there was whole genome methylation in both ‘Fengzao’ and ‘Kyoho’, with varying CHH methylation levels between them. In comparison to ‘Kyoho’, ‘Fengzao’ exhibited higher promoter methylation levels in most genes. Notably, hypermethylation of the *JOX1* promoter resulted in lower expression levels during berry development, ultimately leading to the early ripening of ‘Fengzao’ (Fig. 8e).

## Discussions

### Comparison of DNA methylation in grapes and other plant species

‘Fengzao’ may be the ideal material—as the early-ripening bud mutation of ‘Kyoho’—for studying grape berry development [36, 37]. In the present study, we analyzed whole-genome DNA methylation at six developmental stages of ‘Kyoho’ and five developmental stages of ‘Fengzao’ using WGBS (whole-genome bisulfite sequencing). The coverage and depth of the sequencing were comparable to those of published methylomes in *Arabidopsis* [41], tomato [19], strawberry [26], and sweet orange [18]. The cytosine DNA methylation levels of ‘Kyoho’ and ‘Fengzao’ ranged from 51% to 65% (Fig. 2b) and were significantly higher than the methylation levels in *Arabidopsis* (6%) and tomato (30%) [42–44]. The average DNA methylation level of immature strawberry fruits (i.e., a non-climacteric fruit) was 7.5% [26]. The DNA methylation level of immature sweet orange fruits was 33%, and the genome-wide DNA methylation level was 13% [18]. Therefore, the methylation level of grape is much higher than that of the known climacteric and non-climacteric fruits, suggesting that DNA methylation might play a more important role in grapes.

Average DNA methylation levels were declined during fruit development in tomato and strawberry [19, 23, 26]. However, in sweet orange, the DNA methylation level increased slightly from 13% in immature fruits to 14.5% in mature fruits during the developmental period [18]. The average DNA methylation level of ‘Kyoho’ was highest at the K1 stage (56%) and reduced to the lowest at the K2-K4 stage (52%). For ‘Fengzao’, the highest methylation level was 65% at the F2 stage, and the lowest was 51% at the F1 stage (Fig. 2b, Table S3). The average DNA methylation levels for both ‘Kyoho’ and ‘Fengzao’ did not show a specific trend of increasing or decreasing during berry development, revealing that DNA methylation changes during grape berry development are even more complex.

DNA methylation levels of genes and transposons (TEs) in ‘Kyoho’ gradually increased in the upstream regions of the transcription start site (TSS) and downstream regions of the transcription end site (TES) from the K1 to K6 developmental stages, mainly due to the methylation of CHH, not of CG and CHG (Fig. 3). However, in ‘Fengzao’, the DNA methylation level of genes and TEs was highest in F2 and lowest in F1, showing no specific change in trend during berry development (Fig. 3). The average DNA methylation levels of genes and TEs in sweet orange gradually increased from the first to the last investigated developmental stages, similar to the changes observed in ‘Kyoho’, and the changes were also due to the methylation of CHH [18]. However, in strawberry, the DNA methylation of genes and TEs gradually decreased with the changes in fruit development, contrary to both ‘Kyoho’ and sweet orange [18, 26]. Therefore, for non-climacteric fruits, the DNA methylation levels of genes and TEs in ‘Kyoho’, sweet orange, strawberry, and ‘Fengzao’ showed three different change patterns during fruit development, demonstrating that the regulation of DNA methylation on fruit development has no specific regularity among different plant species. It is uncertain how DNA methylation regulates fruit development and ripening (i.e., positively or negatively) based on the collective results of strawberry, sweet orange, and grape analysis, possibly because DNA methylation in different gene regions (UTR, promoter, or gene body) could have different effects (activation or inhibition) on gene expression. Moreover, the regulatory pathways of fruit development vary in different plant species. Future work should further investigate this phenomenon.

### Hypermethylation (especially CHH) of promoters contributes to grape berry development and ripening

In this study, we found that promoter hypermethylation (especially CHH) may determine grape berry development and ripening. Firstly, by analyzing the methylation levels of genes and their upstream (promoters) and downstream regions, it was found that the methylation levels in the flanking regions of genes were significantly higher than that in the body regions (especially CHH type), and TSS and TES were two obvious demarcation points (Fig. 3), suggesting that hypermethylation may be more active in the flanking regions of genes. As a switch of gene expression, the promoters are highly methylated, which will strongly affect gene expressions. The highly-expressed genes are more likely to be hypermethylated at CHH sequences in their promoter regions [45]. Then, by analyzing the methylation status during the berry development of ‘Kyoho’, we found that the methylation levels of gene promoters gradually increased with the developmental stages, which is consistent with the results in sweet orange [18]. These results indicate that promoter hypermethylation occurs during fruit development and may be one of the factors determining grape ripening. Additionally, the promoter methylation levels of ‘Fengzao’ were generally higher than ‘Kyoho’ during berry development (Fig. 4), and for most of our identified genes, such as those related to secondary metabolites, the methylation levels of promoters in ‘Fengzao’ were higher than that in ‘Kyoho’ (Fig. 6), further proving that promoter hypermethylation may be one of the determinants for the early ripening of ‘Fengzao’. Similar conclusions were also drawn from previous studies on orange and carnation [18, 46], which found that promoter hypermethylation could activate ABA responsive genes and affect fruit ripening [18], and DNA methylation significantly increased in the promoter region of genes during flower senescence in carnation [46]. However, other studies also made different conclusions, indicating that demethylation could activate the expressions of ripening-related genes [19, 21]. Totally speaking, for grapes, it is credible that promoter hypermethylation affects grape berry development and ripening, which mechanisms are complex and need to be further investigated.

The results presented in Fig. 3 demonstrated a gradual increase in methylation levels of ‘Kyoho’ during berry development, with varying trends observed in ‘Fengzao’ and a exceptional increase noted at the F2 stage. Similarly, the results of promoter methylation analysis also showed significant increases at the F2 stage (Fig. 4). This suggests that hypermethylation at the ‘Fengzao’ F2 stage may play an important role in ripening. According to the phenotypes (Fig. 1), ‘Fengzao’ did not show an early-ripening phenotype from F1 to F2 stages, while starting from F2, ‘Fengzao’ ripening stage is gradually advanced, which also indicates that the F2 stage may be crucial. Further GO analysis of genes in cluster 3 (Fig. 4f), which experienced a sharp increase in promoter methylation at the F2 stage, revealed enrichment in transcription factor, protein kinase, peroxidase, and pectinesterase. KEGG enrichment analysis indicated that these genes were associated with peroxidase and SAUR family proteins (Fig. S5). Previous researches have shown a correlation between peroxidase or reactive oxygen species (ROS) and grapes early ripening, with exogenous treatments of hydrogen peroxide and riboflavin promoting early ripening [28, 47]. Collectively, these findings suggest that at the F2 stage, increased methylation levels in the ‘Fengzao’ influence the expressions of transcription factors and kinases, thereby regulating downstream ROS-related genes and promoting early-ripening of ‘Fengzao’.

### Key genes modified by DNA methylation involved in grape berry development

Conjoint analysis of differentially methylated genes (DMGs) and differentially expressed genes (DEGs) in ‘Fengzao’ and ‘Kyoho’ revealed 38 significant genes (Fig. 7) that may serve as novel key regulators in grape berry development and ripening. Among these genes, *JOX1* was further investigated through overexpression in *Arabidopsis* and knockdown in grape berry (Fig. 8), indicating *JOX1* as a negative regulator of grape ripening. *JOX1*, encoding jasmonate-induced oxygenase and belonging to the 2-oxoglutarate-dependent dioxygenase (2OGD) superfamily, is involved in hormone biosynthesis and metabolism in plants [48, 49]. Previous studies have shown the involvement of 2OGD family genes in fruit ripening in tomato [50]. *JOX1* mediates the hydroxylation of jasmonic acid (JA) to 12-OH-JA, an inactive form of JA [51]. In *Arabidopsis*, mutant of *JOX* genes promoted JA accumulation [52]. JA content was found gradually increased during berry development, and exogenous application of JA could promote fruit ripening and induce the expressions of ripening-related genes in both grapes and bananas [29, 53]. So, in theory, *JOX* could negatively regulate fruit ripening by reducing JA levels. In this study, overexpression of grape *JOX1* gene in *Arabidopsis* delayed ripening, while knockdown in grape berry promoted ripening (Fig. 8). These researches confirmed the importance of *JOX*-mediated changes in hormone levels, particularly JA, in fruit ripening. In addition, we also observed the methylation level changes of *JOX1* during grape berry development, which affected its gene expression (Fig. 8a). Previous research revealed the effects of plant hormones on methylation levels and expressions of ripening-related genes in grapes [31]. Most importantly, methylation of genes associated with JA biosynthesis influenced JA levels in plants [45, 54]. So, collectively, we presumed that methylation level of *JOX1* might affect JA levels, thereby influencing grape ripening, which could be further explored in the future.

In addition to *JOX1*, our study also examined other genes, including *HSP20*, *PME*, *PAO4*, *CIPK25*, and *RLK*, that have been modified by DNA methylation and may play a role in grape berry development. HSP20, known as a heat shock protein 20, is a well-established intracellular chaperone protein primarily involved in stress responses [55]. Recent research has also highlighted its significance in fruit development, where it aids in protein folding and degradation to shield them from environmental stressors [56]. This suggests that HSP20 could indirectly influence fruit development and ripening by maintaining the stability of other ripening-regulatory proteins [57]. PME, or pectinesterase, is closely linked to pectin synthesis and directly influences cell wall structure, thereby impacting fruit firmness during ripening [58]. *PAO4*, a member of the polyamine oxidase gene family, is involved in polyamine catabolism and likely plays a role in peach fruit development and ripening [59]. CIPK25, a CBL-interacting serine/threonine-protein kinase, collaborates with CBL (calcineurin B-like calcium sensor) protein to regulate potassium ion transport during grape berry development, influencing fruit quality [60]. RLK, a receptor-like serine/threonine-protein kinase, is engaged in various biological processes such as growth, development, and stress responses [61]. In strawberries, the RLK gene *FaRIPK1* has been found to interact with the ABA receptor to modulate fruit ripening [62]. Therefore, these identified genes are potential regulators involved in grape berry development and ripening.

Overall, in this study, whole-genome bisulfite sequencing was conducted on cultivar ‘Kyoho’ and its early-ripening bud mutant ‘Fengzao’. The results revealed that transposons (TEs) and gene flanking regions exhibited high levels of methylation, particularly in ‘Fengzao’ compared to ‘Kyoho’, attributed to CHH methylation (mCHH). The methylation patterns differed significantly between the two cultivars during berry development, with differentially methylated genes primarily involved in secondary metabolite biosynthesis. Key candidate genes, including the jasmonate-induced oxygenase gene (*JOX1*), were identified as important regulators of berry ripening. This research provides insight into the unique DNA methylation profiles during grape berry development, shedding light on the epigenetic mechanisms governing the early-ripening bud mutant.

## Materials and methods

### Plant materials

Six-year-old grapevines of ‘Kyoho’ and its early-ripening mutant ‘Fengzao’ were grown in the open experimental fields of Henan University of Science and Technology (Luoyang, China) (34.41°N, 112.46°E). The grape berries were collected at different developmental stages based on the E-L system (Table 1) [38]. ‘Fengzao’ were sampled at five developmental stages (F1–F5) from E-L31 (May 24) to E-L35 (June 21) with the berry traits from pea-size to véraison. Correspondingly, ‘Kyoho’ were sampled at six stages (K1–K6), with one stage added between E-L31 and E-L32, because of the longer developmental period of ‘Kyoho’ than ‘Fengzao’. Each sample was taken from three individual grapevines. From each grapevine, two bunches with similar growth patterns and no signs of scars, pests or diseases were collected. The berries with similar size from the middle of each bunch were picked and mixed together. All collected samples were immediately frozen in liquid nitrogen and stored at −80°C until further use.

### Whole-genome bisulfite sequencing (WGBS) and analysis

About 50 g of the stored berries were randomly selected from each sample and mixed together for DNA extraction according to the method described previously [63]. DNA concentration and integrity were detected via 1% (w/v) agarose gel electrophoresis and the NanoDrop2000 spectrophotometer (Thermo Scientific, Waltham, MA, USA). The qualified DNA was submitted to the Anoroad Gene Technology Company (Beijing, China) for subsequent WGBS. In detail, DNA was randomly interrupted by ultrasound to approximately 350 bp, and adaptors were added to the fragments. The DNA fragments were treated with bisulfite, converting unmethylated cytosine (C) into uracil (U). Sequencing was conducted based on the Illumina platform, and the primary DNA methylation libraries were constructed. Following quality control and filtration by removing low-quality reads, clean reads were obtained. The resulting clean reads were compared with the grape reference genome for analysis of whole-genome methylated cytosine (mC) (https://urgi.versailles.inra.fr/files/Vini/Vitis%2012X.2%20annotations/) using Bismark software [64].

PCA (principal components analysis) was conducted with the obtained methylome data for all ‘Kyoho’ and ‘Fengzao’ samples. Transposons (TEs) were identified and annotated as described previously [65]. The top seven types of TEs in terms of abundance were used for further analysis. The methylation of genes and TEs were analyzed separately, and the methylation level was calculated as described previously [19]. The DNA methylation levels of gene promoters (2000 bp upstream of transcription start site ATG) were conducted TCseq cluster analysis using R package vagan with the default criteria based on the methylation values.

### Analysis of differentially methylated cytosine sites (DMCs) and regions (DMRs)

DMCs and DMRs were called using the R package DSS (dispersion shrinkage for sequencing data) [66]. DSS was based on the β-binomial distribution model by performing a strict Wald test. WGBS data was first prepared as the input data for DSS. Next, DMCs were called based on the DML test function from DSS, according to the following steps: (1) calculate the average methylation levels for all cytosine sites; (2) calculate the dispersion levels for all cytosine sites; (3) conduct the Wald test. Cytosine sites with a q value <0.00005 and difference of methylation levels between groups ≥0.1 were retained as DMCs. DMRs were called based on DMCs with DML test function from DSS. Only the regions that showed significant differences between the two compared samples were defined as DMRs. The threshold values for DMRs were set as p-value <0.00005, the difference of mean methylation levels between groups ≥0.1, and containing at least three CpG sites. The IGV 2.15.2 software was used to visually show the methylation status of any selected regions in different samples.

### Analysis of transcriptome data

Previously obtained RNA-sequencing data were used to analyze differentially expressed genes between ‘Kyoho’ and ‘Fengzao’ [37]. The transcriptome data were deposited in the NCBI database (accession numbers SRR1557134 and SRR1558172). The fragments per kilobase of exon model per million mapped fragments (FPKM) values were used to calculate gene expression levels.

### Genetic transformation of *Arabidopsis*

The coding sequence (CDS) of *JOX1* gene was cloned from ‘Kyoho’ grapes and recombined into a pSAK277 vector to produce a *JOX1*-overexpression (*JOX1*-OE) construct. The *JOX1*-OE construct was transformed into the *Arabidopsis* wild-type plants (Col-0) by Agrobacterium-mediated transformation using the floral dipping method [67]. Transgenic plants were first screened using 50 mg/L kanamycin on 1/2 MS (Murashige and Skoog) medium, and then identified by PCR (polymerase chain reaction). The T3 generation of *JOX1*-OE *Arabidopsis* was used for observing phenotype of ripening.

### Transient knockdown of *JOX1* in grape berry

Transiently genetic transformation in ‘Kyoho’ grape berry was conducted based on the pIR system according to the previous study [40]. The coding sequence of *JOX1* gene was reverse-cloned into pIR vector to obtain *JOX1*-RNAi vector. About 30 days after full bloom (DAF), 200 ng of *JOX1*-RNAi plasmid (about 50 μL) was mixed with an equal amount of IL-60-BS helper plasmid. The empty pIR vector was used as control. Spike-stalks of the grape clusters were punctured with a hypodermic needle to make a hole deep into the xylem, and a capillary tube with the plasmid mixture was inserted into the hole. The capillary tube was pulled out until all the plasmid mixture was absorbed by the grape clusters. Five grape clusters were injected for RNAi and control respectively, and injection was performed two times at seven-day intervals. After that, phenotype was observed and samples were collected at about 45 DAF. The effectiveness of *JOX1* knockdown was assessed by qRT-PCR.

## Supplementary Material

Web_Material_uhae285

## Data Availability

The transcriptome data underlying this article are available in the NCBI SRA repository (https://www.ncbi.nlm.nih.gov/sra/) under the BioProject ID: SRR1557134 and SRR1558172. The data generated or analyzed during this study are included in this article, which may be provided by the corresponding author upon reasonable request.
