# Supplementary material for: CHH hypermethylation contributes to the early ripening of grapes revealed by DNA methylome landscape of ‘Kyoho’ and its bud mutant
Source: Hortic Res. 2024 Oct 14;12(1):uhae285. doi: 10.1093/hr/uhae285 (PMC11764089; doi:10.1093/hr/uhae285)
Supplement: Web_Material_uhae285 [file web_material_uhae285.zip › Supplementary files.docx]

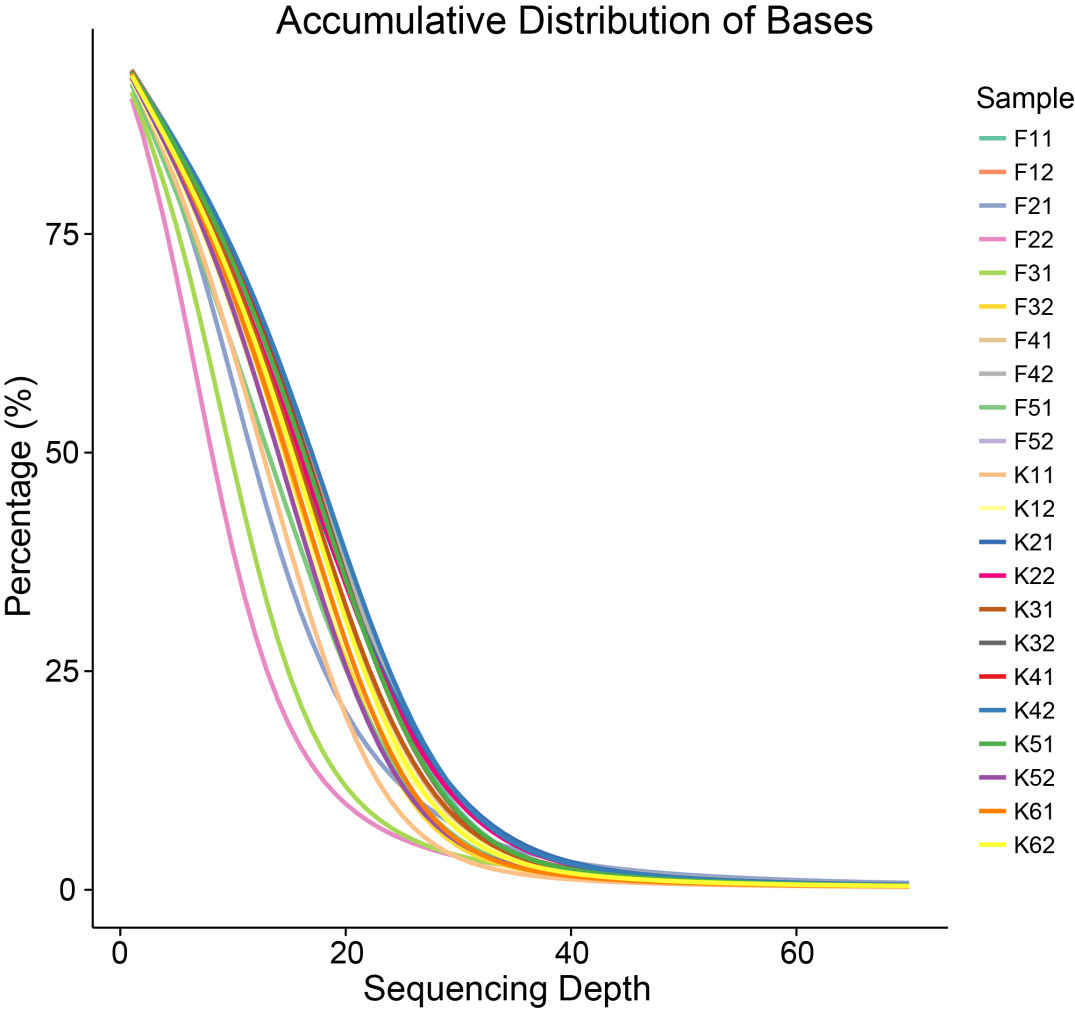


**Figure S1.** The cumulative distribution of cytosine bases under different sequencing depths in each sample.


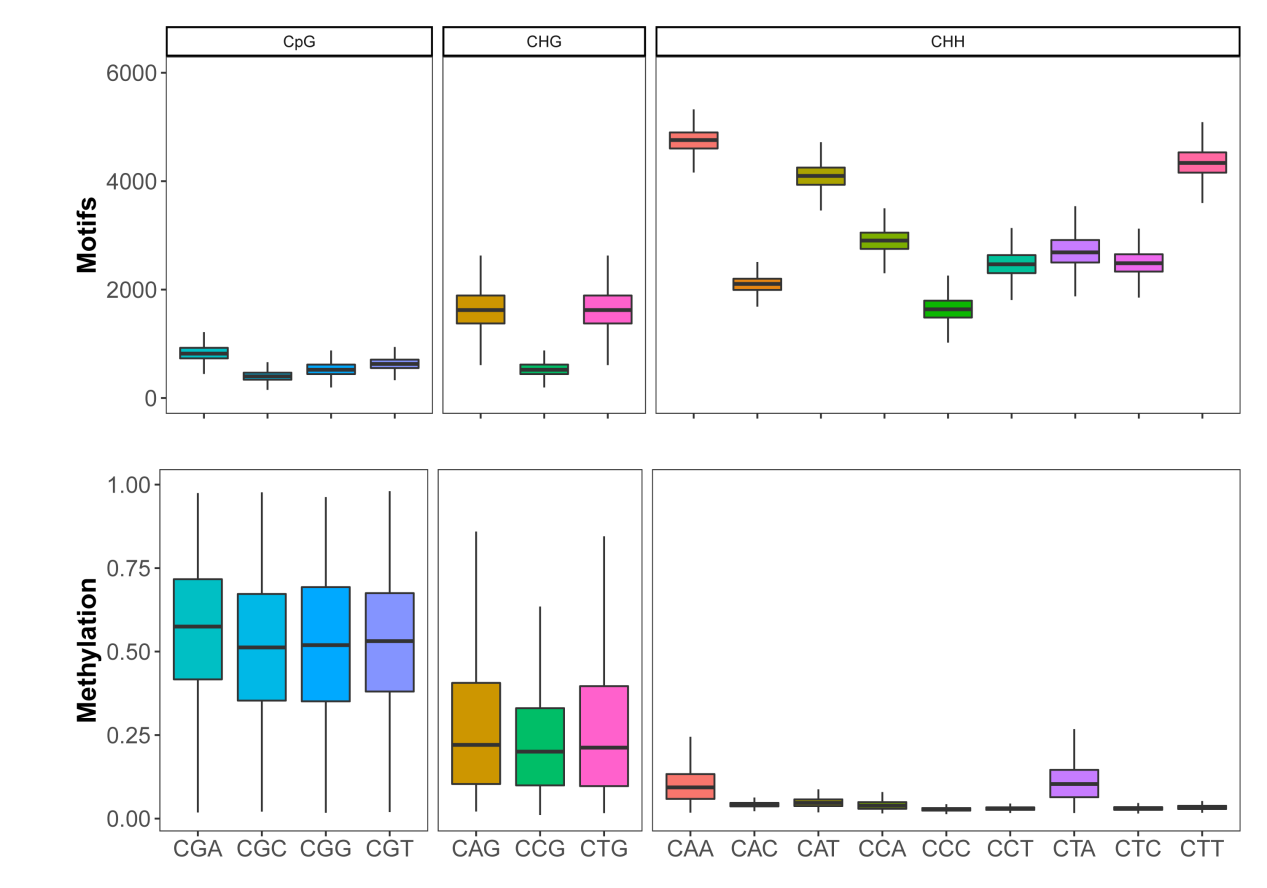


**Figure S2.** The densities (upper panel) and methylation levels (lower panel) for different sequence types of CG, CHG, and CHH in ‘Fengzao’.


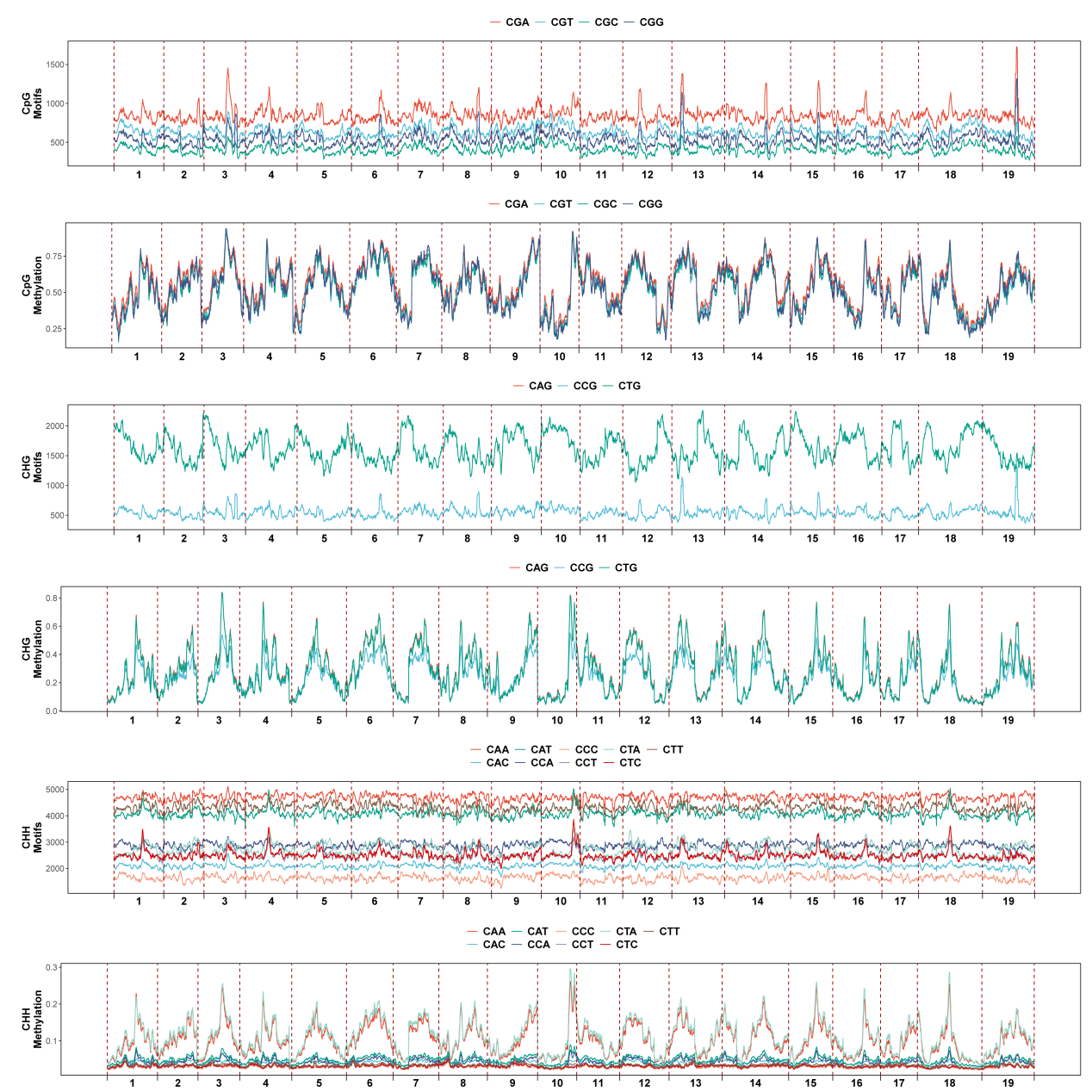


**Figure S3.** The distribution of densities (upper three panels) and methylation levels (lower three panels) for different sequence types of CG, CHG, and CHH across 19 chromosomes in ‘Fengzao’.


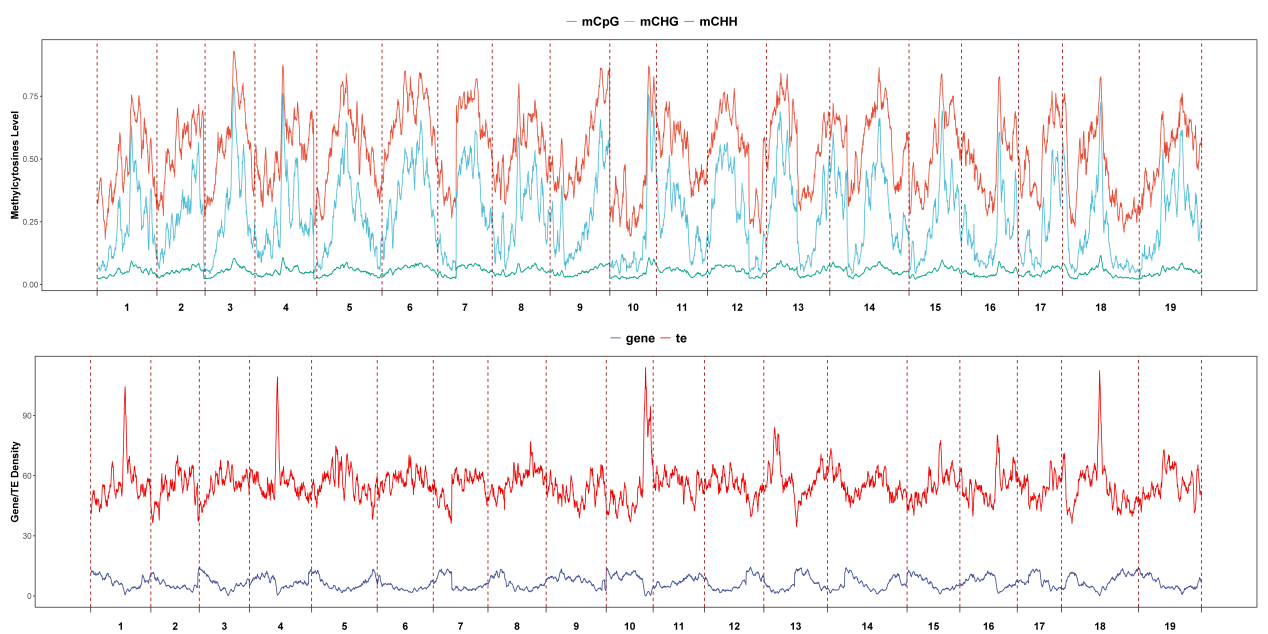


**Figure S4.** The distribution of DNA methylation (upper panel) and densities of genes and transposons (TEs) (lower panel) in three sequence contexts (mCG, mCHG, and mCHH) across chromosomes in ‘Fengzao’.


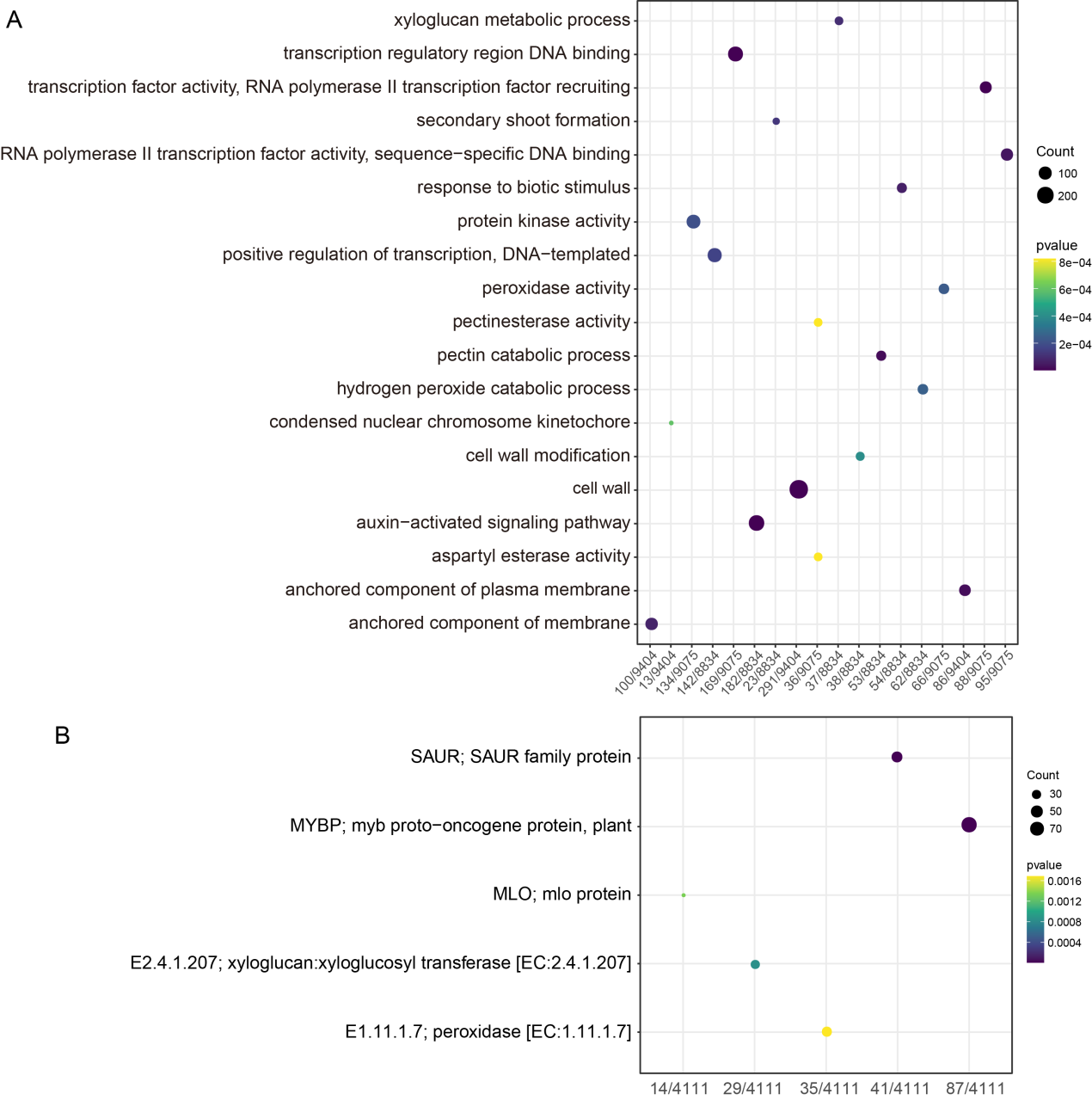


**Figure S5.** GO (A) and KEGG (B) pathway enrichment of genes in cluster 3.


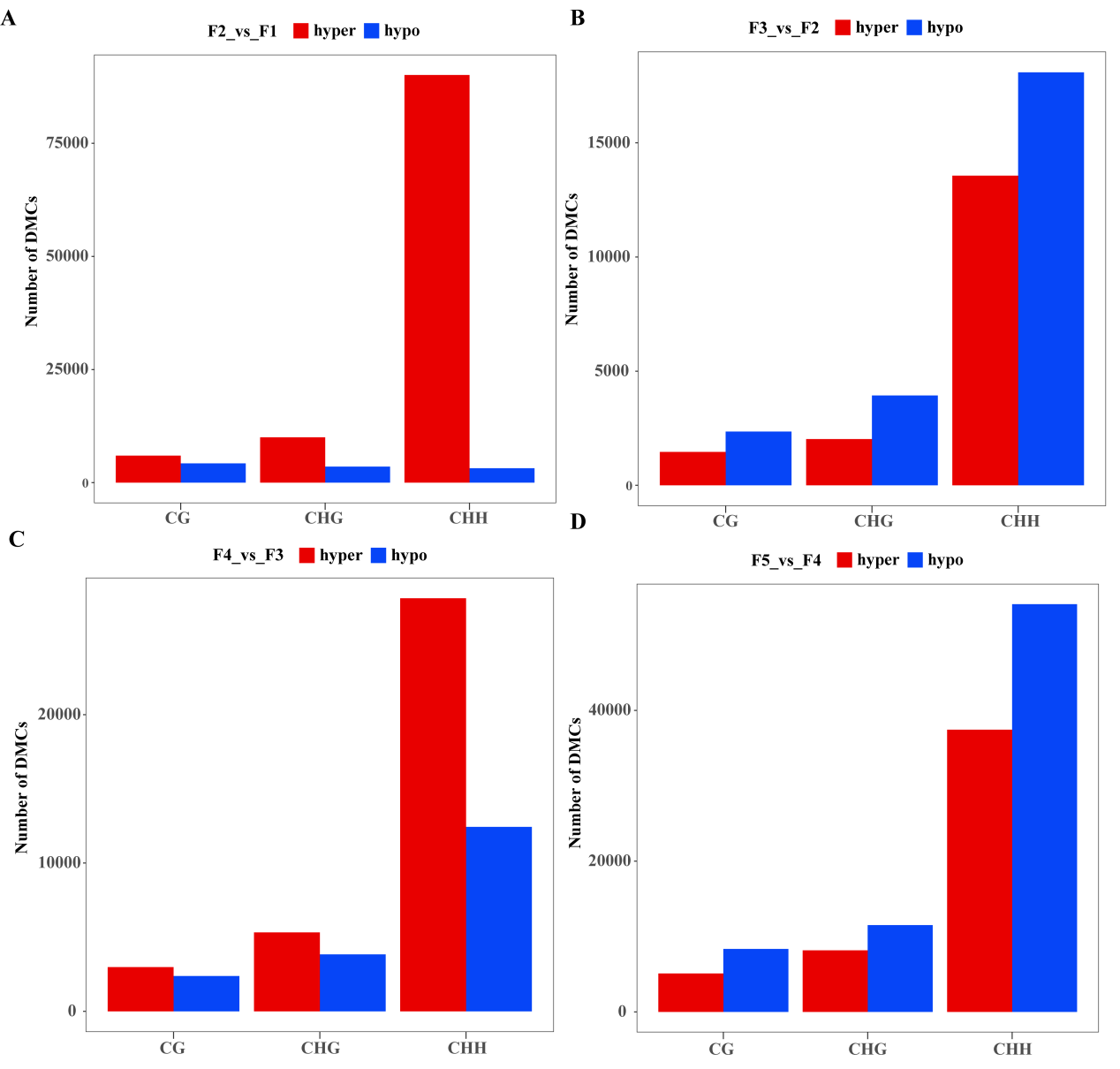


**Figure S6.** Numbers of differentially methylated cytosine sites (DMCs) in different comparisons, namely F2_vs_F1 (A), F3_vs_F2 (B), F4_vs_F3 (C), and F5_vs_F4 (D), in ‘Fengzao’. hyper: hypermethylation. hypo: hypomethylation.


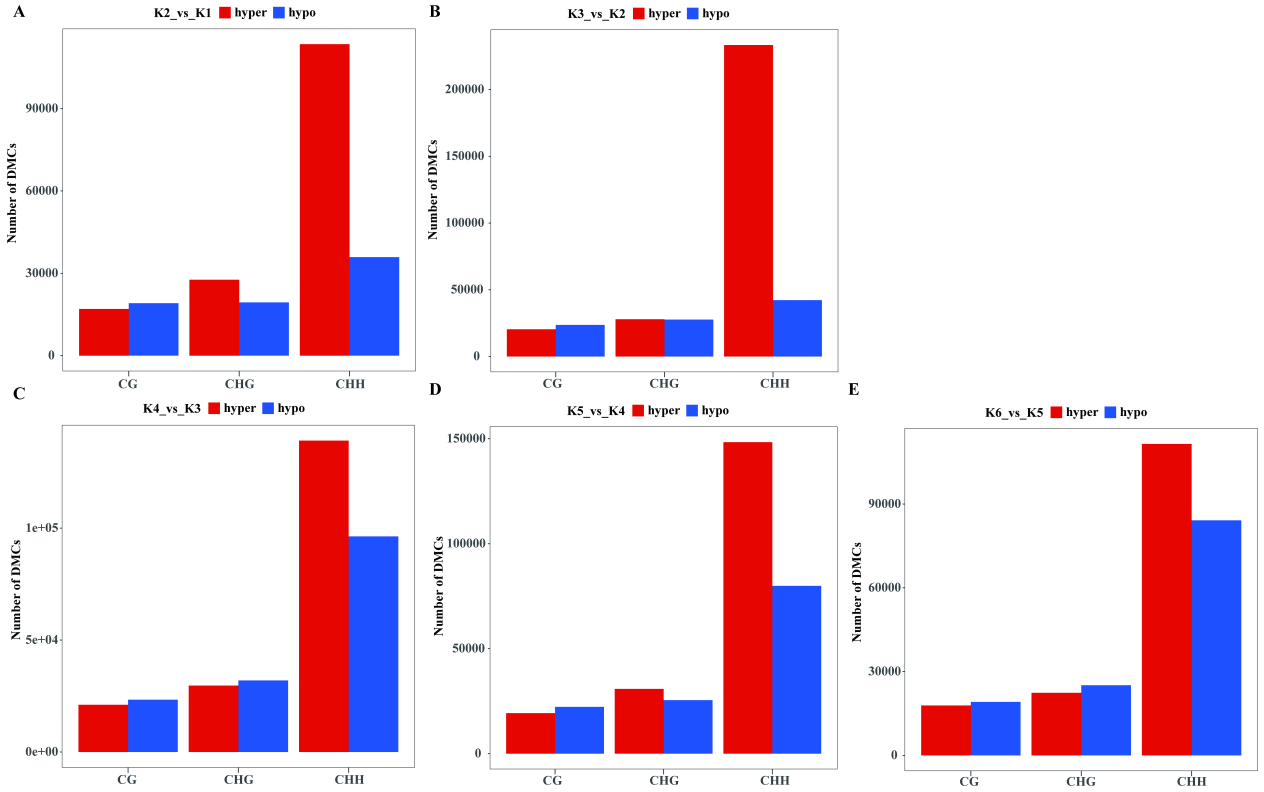


**Figure S7.** Number of DMCs in different comparisons, namely K2_vs_K1 (A), K3_vs_K2 (B), K4_vs_K3 (C), K5_vs_K4 (D), and K6_vs_K5 (E), in ‘Kyoho’. hyper: hypermethylation. hypo: hypomethylation.


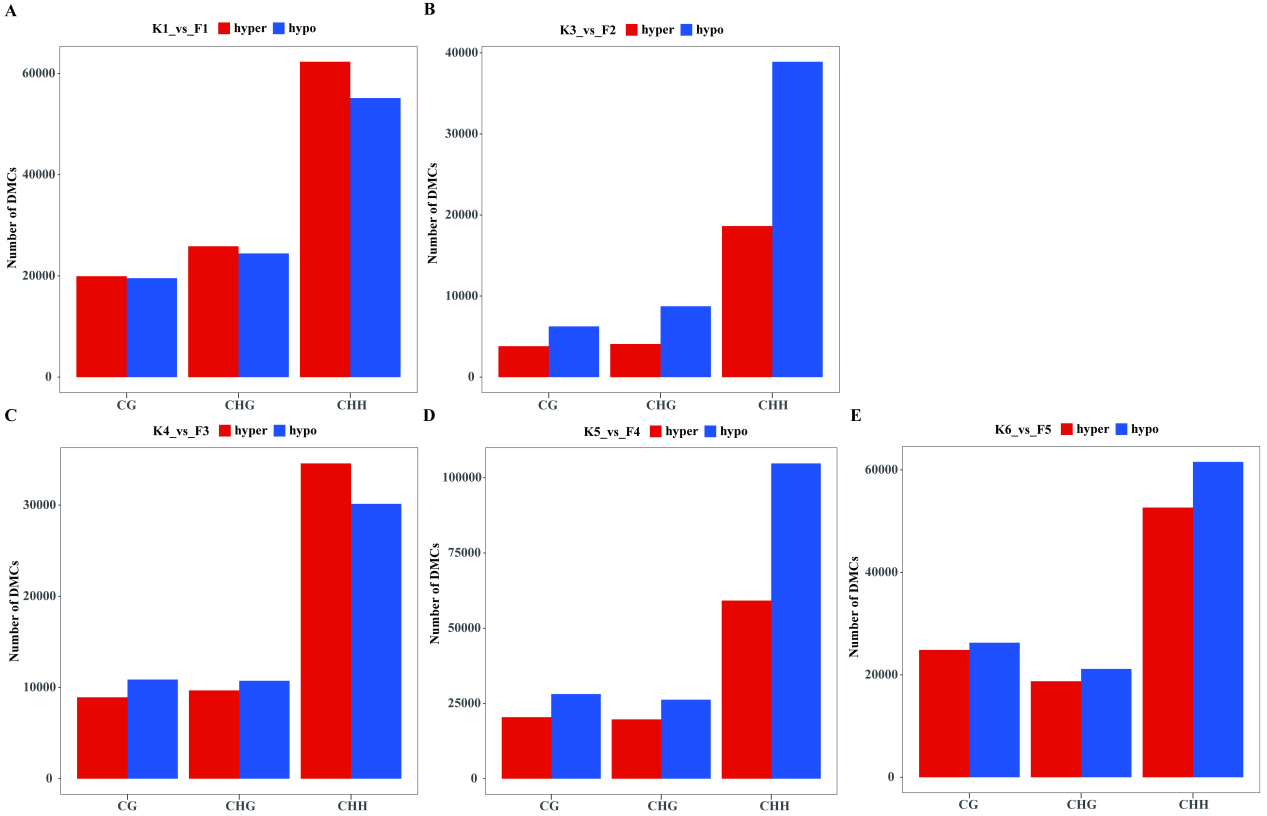


**Figure S8.** Number of DMCs in different comparisons between ‘Fengzao’ and ‘Kyoho’. hyper: hypermethylation. hypo: hypomethylation.


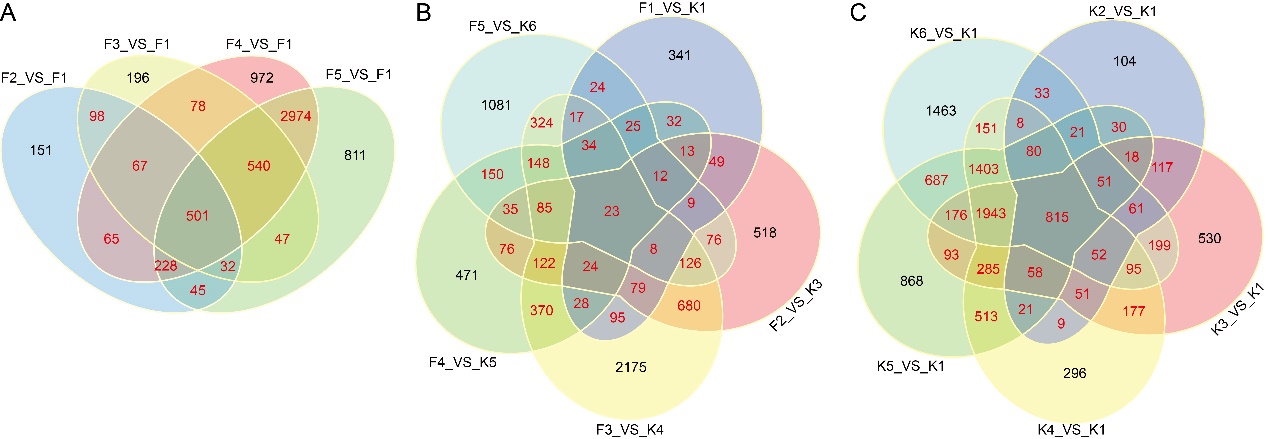


**Figure S9.** Statistics of differentially expressed genes (DEGs) in ‘Fengzao’ and ‘Kyoho’ during berry development.


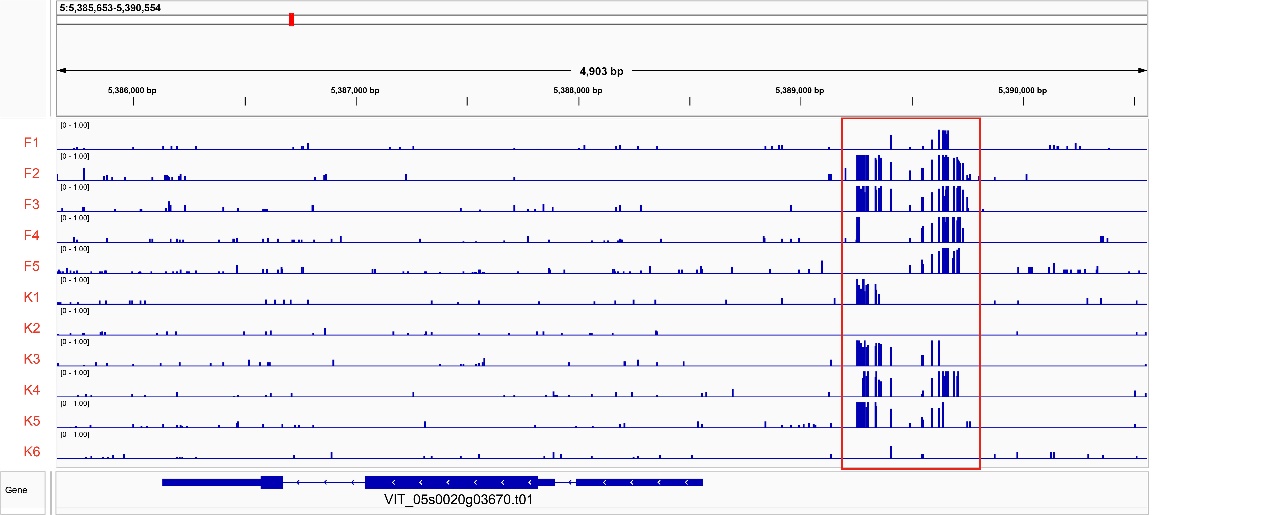


**Figure S10.** Genome browser snapshot showing DNA methylation of VIT_05s0020g03670 in ‘Fengzao’ and ‘Kyoho’.


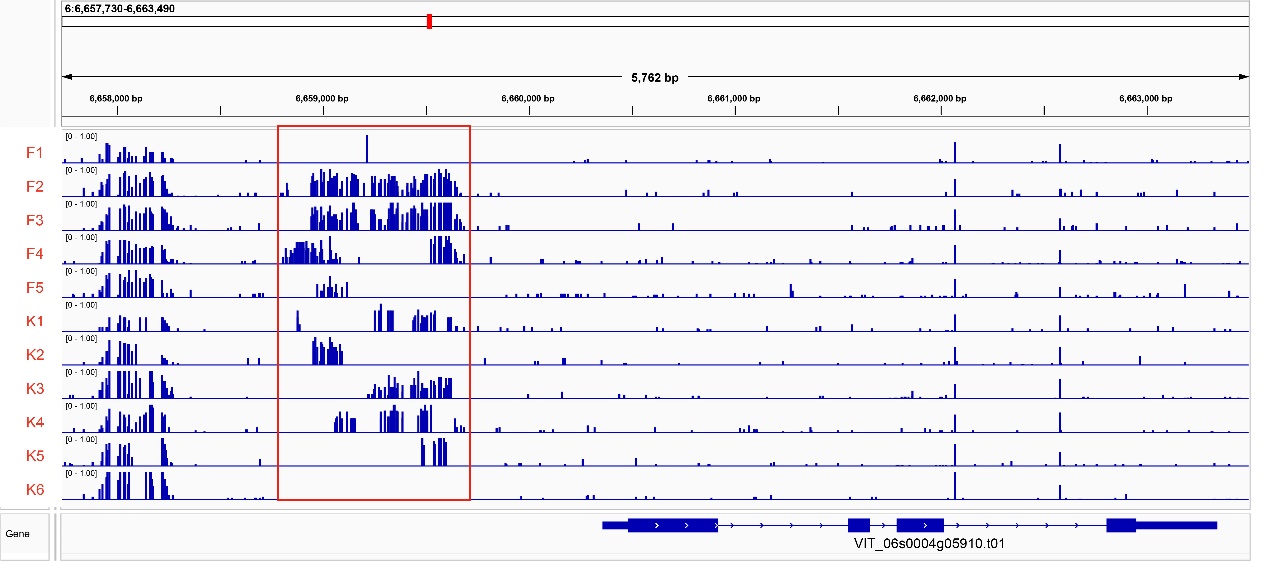


**Figure S11.** Genome browser snapshot showing DNA methylation of VIT_06s0004g05910 in ‘Fengzao’ and ‘Kyoho’.


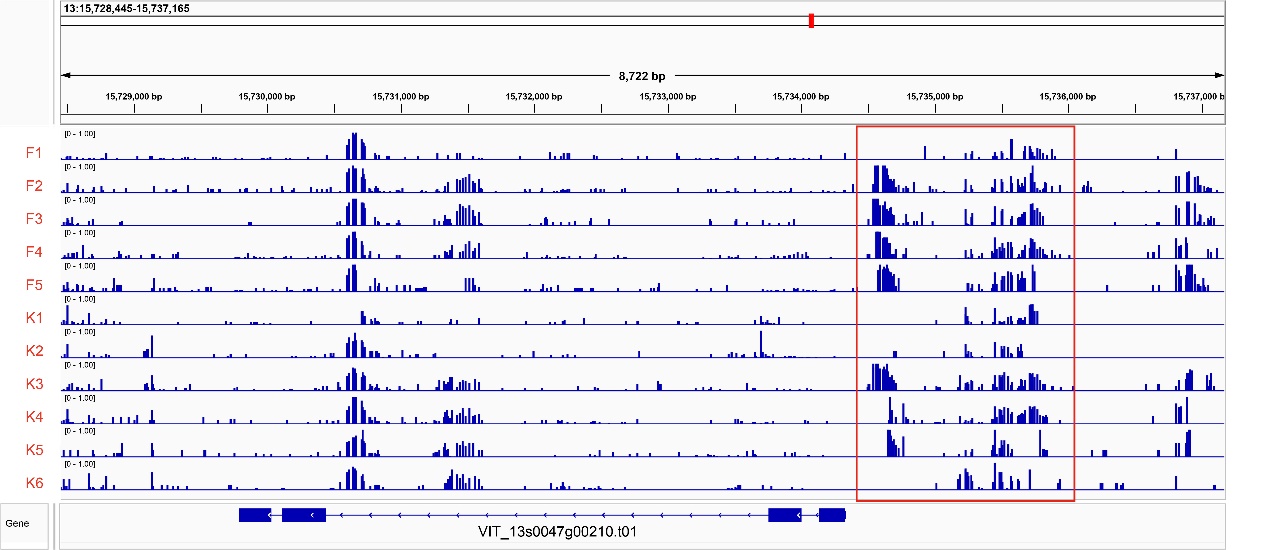


**Figure S12.** Genome browser snapshot showing DNA methylation of VIT_13s0047g00210 in ‘Fengzao’ and ‘Kyoho’.


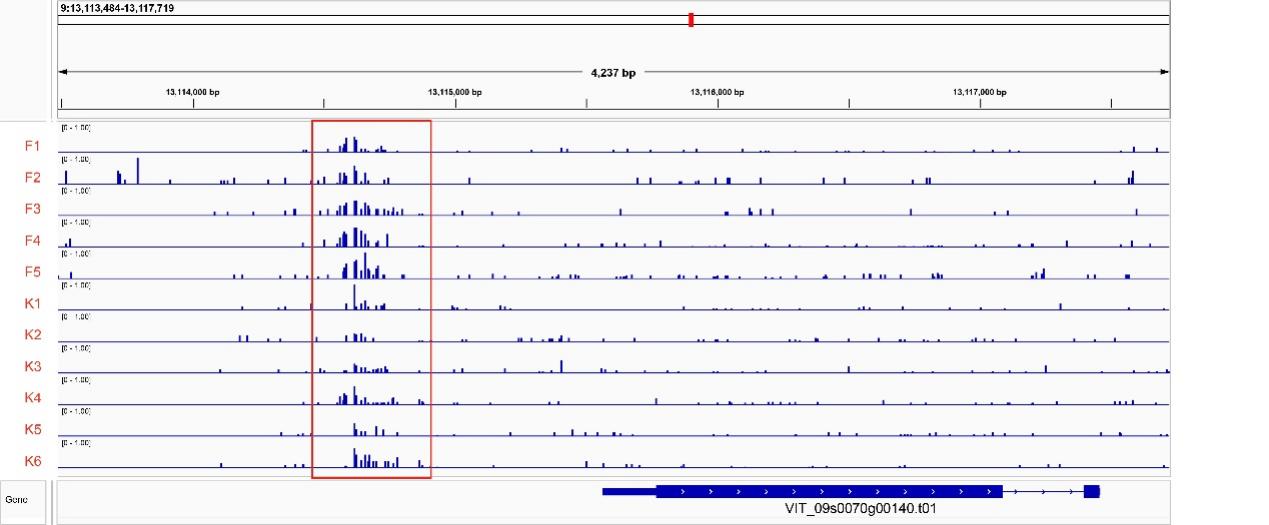


**Figure S13.** Genome browser snapshot showing DNA methylation of VIT_09s0070g00140 in ‘Fengzao’ and ‘Kyoho’.


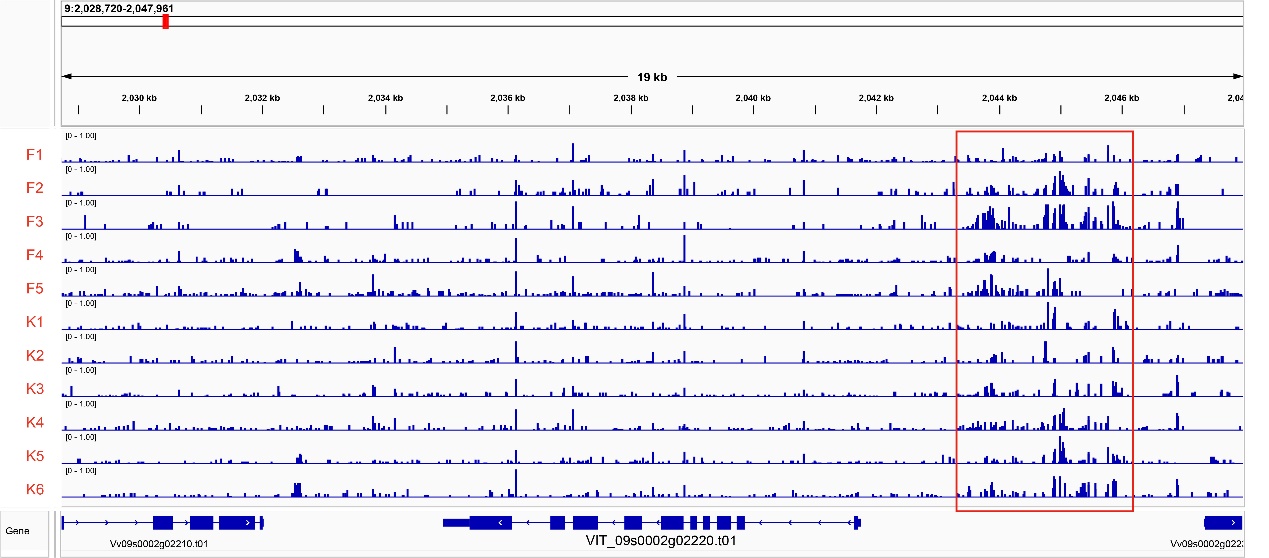


**Figure S14.** Genome browser snapshot showing DNA methylation of VIT_09s0002g02220 in ‘Fengzao’ and ‘Kyoho’.


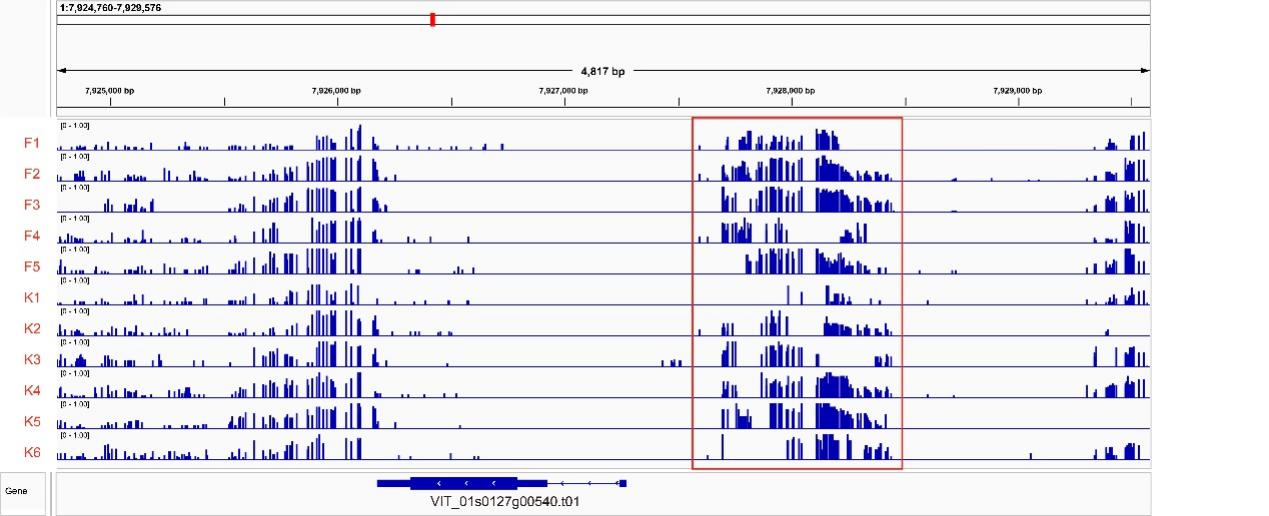


**Figure S15.** Genome browser snapshot showing DNA methylation of VIT_01s0127g00540 in ‘Fengzao’ and ‘Kyoho’.

**
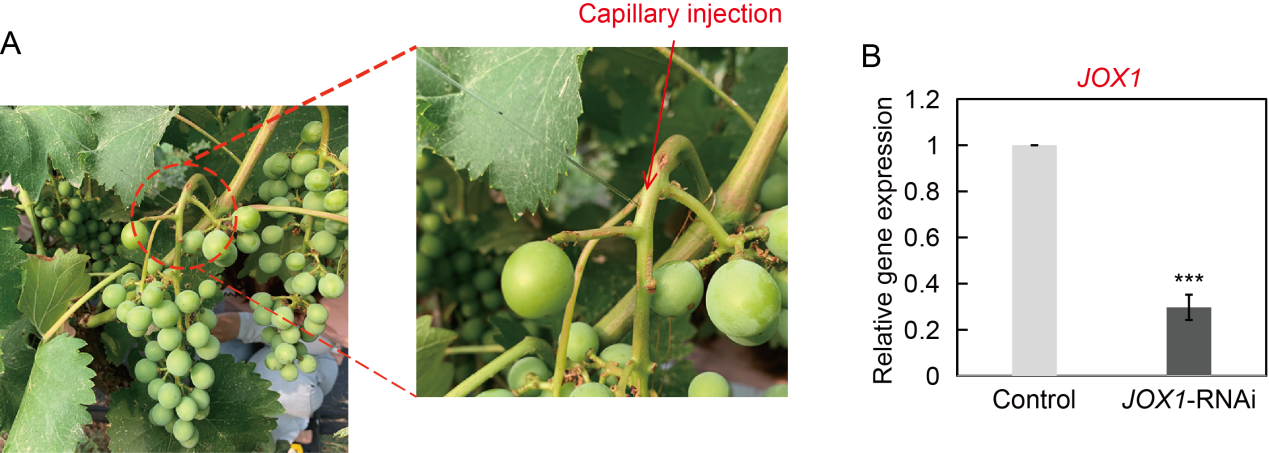
**

**Figure S16.** The schematic image of capillary injection for transient knockdown of *JOX1* gene in grape berry (A) and the relative expression level of *JOX1* in control and *JOX1*-RNAi groups (B). Statistical analysis of the data was conducted using one-way analysis of variance (ANOVA), taking ****P* < 0.001 as significance.

**Table S1. Statistics of sequencing data for all samples**

| **Samples** | **Clean reads** | **Mapped reads** | **Mapped ratio (%)** | **Conversion Rate (%)** |
| --- | --- | --- | --- | --- |
| F11 | 136676342 | 100445748 | 73.49 | 99.03 |
| F12 | 136676342 | 100779382 | 73.74 | 98.91 |
| F21 | 125284258 | 79077238 | 63.12 | 98.54 |
| F22 | 111053876 | 67566342 | 60.84 | 98.95 |
| F31 | 113844816 | 69757538 | 61.27 | 98.90 |
| F32 | 136676342 | 93209116 | 68.2 | 98.95 |
| F41 | 118659712 | 79594184 | 67.08 | 98.93 |
| F42 | 118141482 | 79326114 | 67.15 | 98.17 |
| F51 | 116089848 | 80717618 | 69.53 | 98.23 |
| F52 | 136676342 | 95917294 | 70.18 | 98.95 |
| K11 | 119948640 | 80241268 | 66.9 | 98.88 |
| K12 | 134664648 | 93121946 | 69.15 | 98.97 |
| K21 | 136676342 | 104931300 | 76.77 | 98.93 |
| K22 | 136676342 | 104177588 | 76.22 | 98.87 |
| K31 | 136676342 | 100463602 | 73.5 | 98.94 |
| K32 | 136676342 | 102305516 | 74.85 | 98.87 |
| K41 | 136676342 | 101172000 | 74.02 | 98.95 |
| K42 | 136676342 | 102777716 | 75.2 | 98.99 |
| K51 | 136676342 | 101085614 | 73.96 | 99.04 |
| K52 | 115049756 | 85947526 | 74.7 | 99.07 |
| K61 | 129664894 | 93523818 | 72.13 | 99.07 |
| K62 | 127617038 | 94251388 | 73.85 | 99.09 |

(F: ‘Fengzao’. K: ‘Kyoho’. F1-F5 and K1-K6 indicate different developmental stages for ‘Fengzao’ and ‘Kyoho’. The last number 1, 2 indicate two replicates.)

**Table S2. Statistics of cytosine (C) number and coverage percentage**

| **Sample** | **C_Num** | **CG_Num** | **CHG_Num** | **CHH_Num** | **1X_C** | **5X_C** | **10X_C** |
| --- | --- | --- | --- | --- | --- | --- | --- |
| F42 | 143,222,694 | 10,331,725 | 16,308,159 | 116,582,810 | 91.28 | 62.71 | 26.5 |
| F41 | 142,753,970 | 10,189,731 | 16,172,435 | 116,391,804 | 90.99 | 62 | 25.52 |
| K11 | 142,055,262 | 10,261,104 | 16,153,797 | 115,640,361 | 90.54 | 54.69 | 16.31 |
| K12 | 141,409,469 | 10,240,336 | 16,078,424 | 115,090,709 | 90.13 | 54.09 | 16.12 |
| F11 | 146,032,578 | 10,541,751 | 16,533,601 | 118,957,226 | 93.07 | 68.14 | 31.31 |
| F12 | 145,668,400 | 10,509,071 | 16,478,094 | 118,681,235 | 92.84 | 66.86 | 28.27 |
| F21 | 134,665,838 | 9,724,042 | 15,290,973 | 109,650,823 | 85.83 | 37.72 | 9.95 |
| F22 | 124,985,163 | 8,970,817 | 14,195,649 | 101,818,697 | 79.66 | 21.99 | 4.65 |
| K51 | 144,946,285 | 10,474,691 | 16,418,471 | 118,053,123 | 92.38 | 65.36 | 27.15 |
| K52 | 142,264,644 | 10,281,841 | 16,156,008 | 115,826,795 | 90.67 | 56.07 | 17.01 |
| K21 | 142,915,102 | 10,361,038 | 16,244,822 | 116,309,242 | 91.09 | 60.24 | 23.03 |
| K22 | 143,650,911 | 10,413,581 | 16,319,442 | 116,917,888 | 91.56 | 61.98 | 24.72 |
| K61 | 144,252,056 | 10,391,743 | 16,333,467 | 117,526,846 | 91.94 | 62.33 | 23.17 |
| K62 | 143,502,041 | 10,346,546 | 16,252,851 | 116,902,644 | 91.46 | 60.68 | 20.66 |
| F51 | 140,751,094 | 10,081,593 | 16,033,539 | 114,635,962 | 89.71 | 54.74 | 16.85 |
| F52 | 143,108,132 | 10,264,084 | 16,188,904 | 116,655,144 | 91.21 | 63.69 | 25.57 |
| K42 | 144,853,432 | 10,475,081 | 16,407,920 | 117,970,431 | 92.32 | 65.53 | 27.79 |
| K41 | 145,146,709 | 10,479,758 | 16,430,069 | 118,236,882 | 92.51 | 65.68 | 27.73 |
| F32 | 142,805,800 | 10,216,767 | 16,133,352 | 116,455,681 | 91.02 | 62.03 | 22.77 |
| F31 | 130,516,728 | 9,388,796 | 14,877,839 | 106,250,093 | 83.19 | 28.18 | 5.27 |
| K32 | 145,135,731 | 10,478,626 | 16,422,971 | 118,234,134 | 92.5 | 65.64 | 26.77 |
| K31 | 143,905,060 | 10,410,643 | 16,326,871 | 117,167,546 | 91.72 | 61.8 | 23.42 |

(C_Num: number of identified C in whole genome. CG/CHG/CHH_Num: number of identified CG/CHG/CHH in whole genome. 1/5/10X_C: the proportion of C with coverage ≥1X, 5X or 10X to the total number of C on the genome.)

**Table S3.** **Total cytosine methylation levels at different developmental stages in ‘Fengzao’ and ‘Kyoho’**

| **Sample** | **Methylation level** | **Sample** | **Methylation level** |
| --- | --- | --- | --- |
| F1 | 0.5084 | K1 | 0.5558 |
|  |  | K2 | 0.5215 |
| F2 | 0.6550 | K3 | 0.5202 |
| F3 | 0.5882 | K4 | 0.5171 |
| F4 | 0.5460 | K5 | 0.5436 |
| F5 | 0.5383 | K6 | 0.5479 |
